# Divalent nanobodies to platelet CLEC-2 can serve as agonists or antagonists

**DOI:** 10.1038/s42003-023-04766-6

**Published:** 2023-04-07

**Authors:** Joanne C. Clark, Eleyna M. Martin, Luis A. Morán, Ying Di, Xueqing Wang, Malou Zuidscherwoude, Helena C. Brown, Deirdre M. Kavanagh, Johan Hummert, Johannes A. Eble, Bernhard Nieswandt, David Stegner, Alice Y. Pollitt, Dirk-Peter Herten, Michael G. Tomlinson, Angel García, Steve P. Watson

**Affiliations:** 1 Institute of Cardiovascular Sciences, Level 1 IBR, College of Medical and Dental Sciences, University of Birmingham, Edgbaston, Birmingham B15 2TT UK; 2 Centre of Membrane Proteins and Receptors (COMPARE), The Universities of Birmingham and Nottingham, The Midlands, UK; 3 Centre for Research in Molecular Medicine and Chronic Diseases (CIMUS), Universidade de Santiago de Compostela, and Instituto de Investigación Sanitaria de Santiago (IDIS), Santiago de Compostela, Spain; 4 School of Biosciences, University of Birmingham, Edgbaston, Birmingham B15 2TT UK; 5 Institute of Experimental Biomedicine I, University Hospital and Rudolf Virchow Center for Integrative and Translational Bioimaging, University of Würzburg, Würzburg, Germany; 6 Department of Biochemistry, University of Oxford, South Parks Road, Oxford, OX1 QU3 UK; 7 Institute for Physiological Chemistry & Pathobiochemistry, University of Münster, Waldeyerstraße 15, 48149 Münster, Germany; 8 Institute for Cardiovascular and Metabolic Research, School of Biological Sciences, University of Reading, Reading, RG6 6AS UK

**Keywords:** Platelets, Cell signalling, Membrane proteins, Fluorescence imaging

## Abstract

CLEC-2 is a target for a new class of antiplatelet agent. Clustering of CLEC-2 leads to phosphorylation of a cytosolic YxxL and binding of the tandem SH2 domains in Syk, crosslinking two receptors. We have raised 48 nanobodies to CLEC-2 and crosslinked the most potent of these to generate divalent and tetravalent nanobody ligands. Fluorescence correlation spectroscopy (FCS) was used to show that the multivalent nanobodies cluster CLEC-2 in the membrane and that clustering is reduced by inhibition of Syk. Strikingly, the tetravalent nanobody stimulated aggregation of human platelets, whereas the divalent nanobody was an antagonist. In contrast, in human CLEC-2 knock-in mouse platelets, the divalent nanobody stimulated aggregation. Mouse platelets express a higher level of CLEC-2 than human platelets. In line with this, the divalent nanobody was an agonist in high-expressing transfected DT40 cells and an antagonist in low-expressing cells. FCS, stepwise photobleaching and non-detergent membrane extraction show that CLEC-2 is a mixture of monomers and dimers, with the degree of dimerisation increasing with expression thereby favouring crosslinking of CLEC-2 dimers. These results identify ligand valency, receptor expression/dimerisation and Syk as variables that govern activation of CLEC-2 and suggest that divalent ligands should be considered as partial agonists.

## Abstract (web summary)

Fluorescence correlation spectroscopy on crosslinked nanobodies tested on C-type lectin-like receptor 2 (CLEC-2) show different outcomes for divalent and tetravalent nanobody ligands on CLEC-2 clustering, also dependent on CLEC-2 expression levels.

## Introduction

Platelets are targets for treatment and prevention of cardiovascular disease pathophysiology, but current anti-platelet treatments carry a risk of excessive bleeding. There is therefore an unmet need for a new class of anti-platelet agent that preserves haemostasis. The platelet glycoprotein receptor C-type lectin-like receptor 2 (CLEC-2) is considered such a target due to its critical role in thrombo-inflammatory disease but negligible role in haemostasis in humans^1,2^.

The endogenous ligand for CLEC-2 is the transmembrane protein podoplanin, which is up-regulated on macrophages and stromal cells at sites of inflammation in human and mice, and is present at high levels on tumour cells^1,3,4^. However, podoplanin is not expressed in the vasculature. In mice, podoplanin has been shown to drive thrombosis in the venous system in models of thrombo-inflammatory disease and proposed to mediate cancer metastasis^5–8^.

CLEC-2 is a type II membrane protein with an extracellular C-type lectin-like domain, a single transmembrane helix and a short cytosolic region. The cytosolic tail contains a single tyrosine residue in a conserved motif that represents half of an immunoreceptor-tyrosine-based-activation-motif (ITAM) and is termed a hemITAM^9^. Clustering of CLEC-2 leads to tyrosine phosphorylation of the hemITAM which allows crosslinking of two phosphorylated CLEC-2 receptors by the tandem SH2 domains in spleen tyrosine kinase (Syk). This initiates a signalling cascade that culminates in phospholipase Cγ2 and platelet activation^10^.

The clustering of CLEC-2 is believed to be facilitated by its ability to dimerise in the membrane^11^. CLEC-2 has been shown to be expressed as a dimer in transfected cell lines by co-precipitation and bioluminescence resonance energy transfer and as a mixture of monomers and dimers in platelets using a covalent crosslinker^12–14^. The CLEC-2 C-type lectin-like domain in isolation is exclusively monomeric and the crystal structure shows no evidence of dimerisation, however, the recombinant glycosylated full extracellular region of CLEC-2 containing the stalk forms a dimer^13,15–17^.

The snake venom toxin rhodocytin, which is composed of multimers of α and β subunits, and podoplanin-expressing cells drive powerful activation of human and mouse platelets, whereas their recombinant monovalent proteins are inactive^12,18,19^. Although this demonstrates that multivalency is critical for activation, we have only a rudimentary understanding of the relationship between clustering and signal strength and the degree to which this is governed by ligand valency. Further, there is evidence for a differential dependency on clustering between human and mouse platelets in mediating activation. The F(ab’)_2_ fragment of the monoclonal antibody (mAb) AYP1 is unable to induce aggregation in human platelets, whereas the mAb to CLEC-2 INU1 stimulates activation of mouse platelets^20–22^. It is not known if this is due to a difference between the two antibodies, human and mouse forms of CLEC-2 or between human and mouse platelets. Fab fragments of INU1 do not induce activation of mouse platelets in vitro confirming the dependency on clustering^21,22^.

In the present study, we have raised a panel of nanobodies against the C-type lectin-like domain of CLEC-2 and crosslinked the most potent of these to generate divalent and tetravalent ligands in order to investigate the dependency on valency for activation. The novel nanobody-based ligands have been tested in human platelets, human CLEC-2 knock-in *(hCLEC1b*^*KI*^*)* mouse platelets and transfected cell lines. The results demonstrate that divalent ligands can act as agonists or antagonists against human CLEC-2, and highlight receptor expression as one variable that governs the response.

## Results

### Generation and characterisation of nanobody-based ligands to CLEC-2

**Fig. 1:**
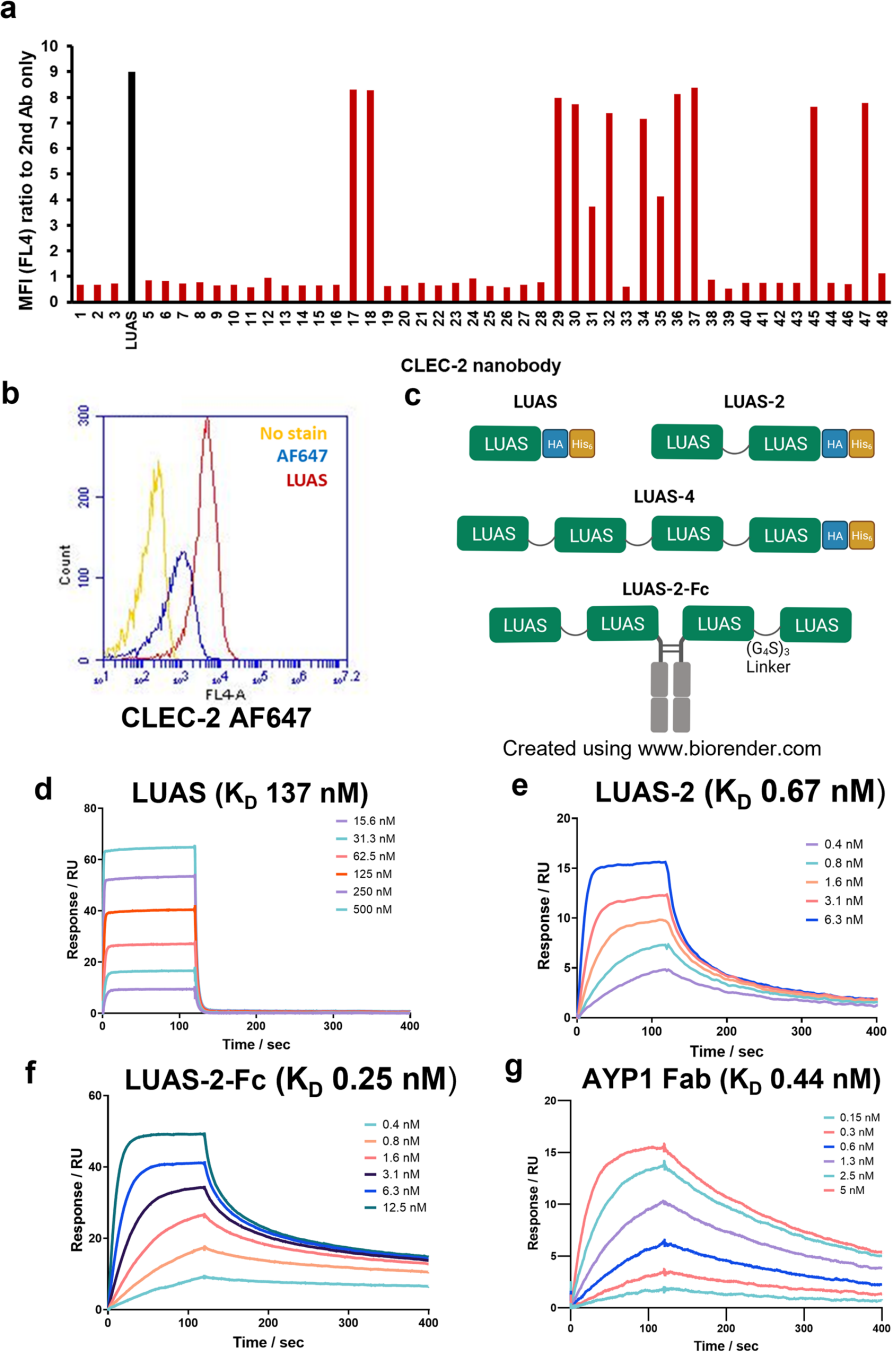
Characterisation of CLEC-2 ligands. **a** Representative flow cytometry data showing binding of CLEC-2 nanobodies (7 nM) to human platelets measured with Alexa Fluor-647 anti-His tag antibody secondary staining (5 μg/ml) to the his-tag on the nanobodies. Data presented as MFI normalised for MFI of secondary staining alone (a.u.). The strongest binder nanobody LUAS (Black bar) is highlighted (*n* = 3 biologically independent experiments). **b** Representative fluorescence intensity histogram overlays showing binding of LUAS (red) (7 nM) to platelets measured with anti-Alexa Fluor-647 (AF647) anti-His tag antibody secondary staining (5 μg/ml) to the his-tag on the nanobodies. Yellow histogram shows platelets with no stain and blue histogram shows secondary staining alone. **c** Schematic representation of the CLEC-2 monovalent, LUAS, divalent LUAS-2 and tetravalent LUAS-4 and LUAS-2-Fc nanobody ligands. LUAS subunits are connected with (G_4_S)_3_ amino acid linkers and have HA and His_6_ tags. The tetravalent LUAS-2-Fc was made by dimerisation of divalent LUAS-2 with a mouse Fc domain (IgG2a). **d**–**g** Representative SPR sensograms showing the binding of LUAS, LUAS-2, LUAS-2-Fc and AYP1 Fab to an immobilised surface of recombinant His_6_-tagged CLEC-2 (residues 55-229). The binding affinities, *K*_D_, were determined as 137 ± 7, 0.67 ± 0.09, 0.25 ± 0.09 and 0.44 ± 0.03 nM for LUAS, LUAS-2, LUAS-2-Fc and AYP1 Fab, respectively. Values were calculated by kinetic analysis using a global fitting model within the Biacore T200 evaluation software (*n* = 3 biologically independent experiments).

In order to develop a high-affinity reagent of known valency to CLEC-2, we raised a series of nanobodies to the C-type lectin-like domain of human CLEC-2 (rec-hCLEC-2: residues 55-229) as described in the Supplementary Methods Section. In total, 48 nanobodies were raised and tested for their ability to bind to CLEC-2 in human platelets by flow cytometry using an Alexa Fluor-647 anti-His tag antibody (Fig. 1a). 13 nanobodies from 5 different subclasses exhibited potent binding to human platelets as illustrated for one of the most potent nanobodies, termed LUAS (Fig. 1b). Based on this potency, LUAS was selected for crosslinking using a (GGGGS)_3_ linker sequence to form a divalent (LUAS-2) and tetravalent (LUAS-4) ligand as illustrated in Fig. 1c. An additional tetravalent ligand was generated by addition of a mouse Fc domain (IgG2a, which does not bind to FcγRIIA) at the C-terminus of divalent LUAS-2, termed LUAS-2-Fc (Fig. 1c).

The ability of LUAS-2 and LUAS-2-Fc to bind to CLEC-2 was compared to the monovalent parent nanobody, LUAS, using surface plasmon resonance (SPR). Varied concentrations of LUAS flowed over a surface of recombinant-hCLEC-2 gave a calculated *K*_D_ of 137 ± 7 nM (Fig. 1d). The dimerisation (LUAS-2) and tetramerisation (LUAS-2-Fc) of LUAS increased the *K*_D_ to 0.67 ± 0.09 nM and to 0.25 ± 0.09 nM, respectively (Fig. 1e, f). The increase in *K*_D_ of the multivalent nanobody is due to avidity as a result of binding to 2 and up to 4 CLEC-2 molecules. The *K*_D_ of LUAS-2 is comparable to that of the Fab of AYP1 of 0.44 ± 0.03 nM (Fig. 1g).

### Divalent and tetravalent nanobodies cluster CLEC-2 in a membrane

The ability of divalent LUAS-2 and tetravalent LUAS-4 to cluster CLEC-2 on the cell surface was investigated using confocal microscopy and fluorescence correlation spectroscopy (FCS) in combination with photon counting histogram (PCH) analysis. FCS is a single molecule microscopy technique that measures fluorescence intensity fluctuations generated by fluorescently-tagged proteins diffusing through a stationary confocal volume (~0.2 fl) to generate information on diffusion rates and molecular brightness^23,24^. The fluorescence intensity is proportional to the number of fluorescently tagged molecules within a protein complex. The FCS confocal volume was determined by measurement of the axial and lateral radii (Supplementary Fig. 1a) and corresponded with previous studies^25,26^.

**Fig. 2:**
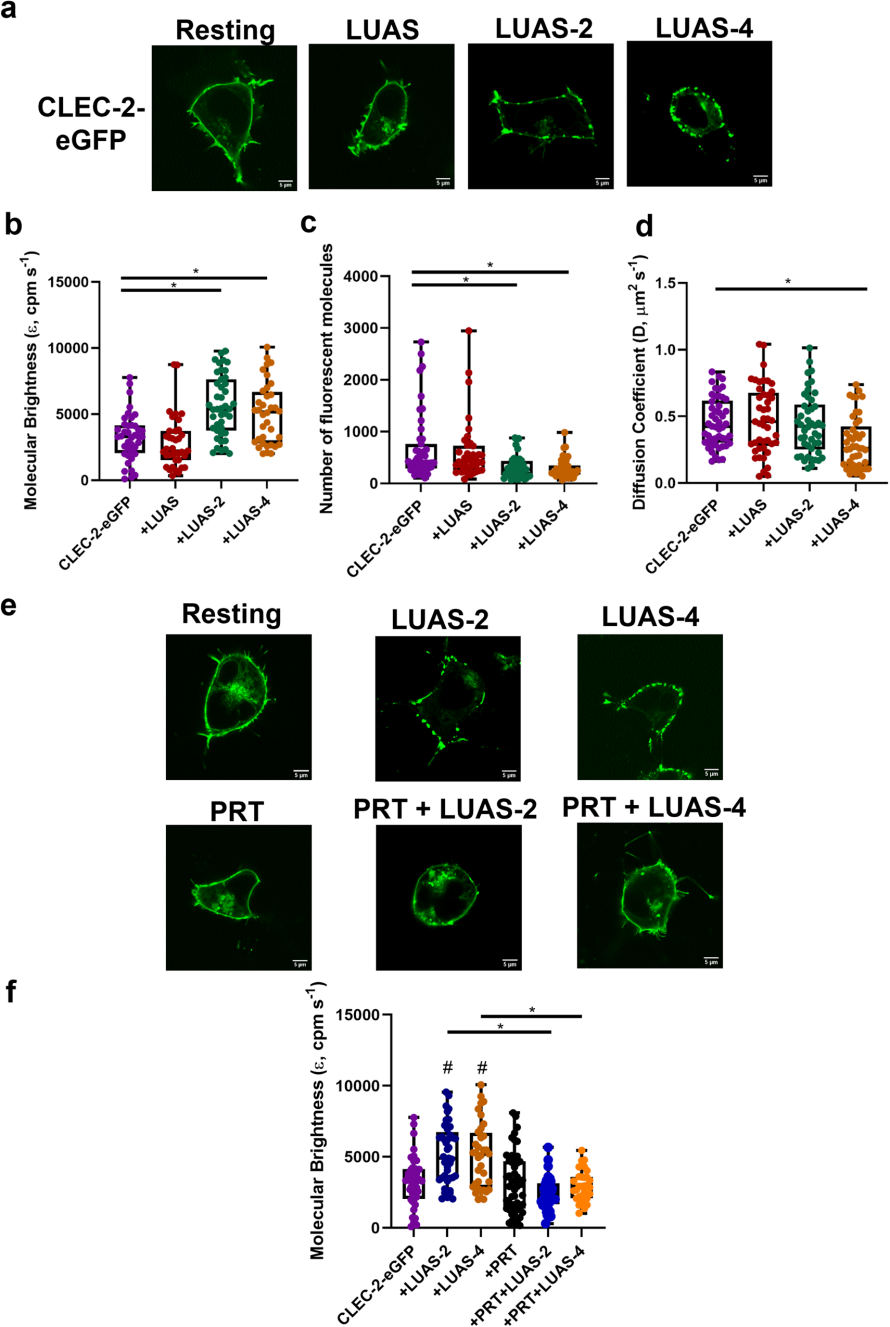
Multivalent CLEC-2 nanobodies cause clustering of CLEC-2 and Syk plays a critical role. **a** Representative confocal microscopy images showing membrane localisation of CLEC-2-eGFP under basal condition and treated with LUAS (10 nM), LUAS-2 (10 nM) and LUAS-4 (10 nM) for 60 min in transfected HEK293T cells (field of view = 52 × 52 μm) (scale bar = 5 μm). Box plots showing the effect of LUAS (10 nM) LUAS-2 (10 nM) and LUAS-4 (10 nM) on CLEC-2-eGFP **b** molecular brightness (ε, counts per molecule per second, cpm s^−1^), **c** number of fluorescent molecules within the confocal volume and **d** diffusion coefficient. FCS measurements were taken in 45–50 cells (*n* = 3 biologically independent experiments). **e** Representative confocal microscopy images showing membrane localisation of CLEC-2-eGFP resting and treated with LUAS-2 (10 nM), LUAS-4 (10 nM), PRT-060318 (10 μM), PRT (10 μM) + LUAS-2 (10 nM) and PRT (10 μM) + LUAS-4 (10 nM) in transfected HEK293T cells (field of view = 52 × 52 μm) (scale bar = 5 μm). Samples were pre-treated with PRT for 45 min prior to agonist addition for 60 min. **f** Box plot showing the effect of the treatments on the molecular brightness (cpm s^−1^) of CLEC-2-eGFP. FCS measurements were taken in 31–49 cells (*n* = 3 biologically independent experiments). For all box plots, centre lines represent the median; box limits indicate the 25th and 75th percentiles and whiskers extend to minimum and maximum points. Significance was measured with Kruskal-Wallis with Dunn’s post-hoc where *P* ≤ 0.05. In (**f**) # = significance compared to CLEC-2 alone (no ligand).

To perform these studies, we labelled the cytosolic N-terminus of CLEC-2 via a linker with eGFP. Plasma membrane localisation of CLEC-2-eGFP was confirmed by confocal microscopy with CLEC-2 showing a uniform distribution on the surface of the HEK293T cells (Fig. 2a). Count rate (average fluorescence intensity) was used to select cells with a similar level of expression of CLEC-2. Measurement of time-dependent fluorescence intensity fluctuations by FCS generated autocorrelation curves and PCHs for determination of diffusion coefficients and molecular brightness, respectively. FCS measurements of CLEC-2 transfected HEK293T cells showed that divalent LUAS-2 and tetravalent LUAS-4 but not monovalent LUAS caused a significant increase in the molecular brightness of CLEC-2-eGFP and a reduction in the number of species, confirming clustering of CLEC-2 (Fig. 2b, c). LUAS-4 also decreased the diffusion coefficient of CLEC-2 (Fig. 2d) which is likely due to the combined size of the reagent and formation of large clusters of CLEC-2.

We next investigated the effect of the Syk inhibitor PRT-060318 on the nanobody-induced clustering of CLEC-2. The discrete patches of CLEC-2-eGFP induced by LUAS-2 and LUAS-4 observed in the confocal images (Fig. 2a, e) were inhibited in the presence of PRT-060318 as was the increase in molecular brightness of CLEC-2-eGFP (Fig. 2f). These observations demonstrate a critical role for the tyrosine kinase in cluster formation by the two ligands. In the FCS molecular brightness recordings, there was an absence of lower molecular brightness values with tetravalent LUAS-4-treated cells relative to the LUAS-2-treated cells in the presence of PRT-060318, consistent with its higher valency.

These results demonstrate that divalent LUAS-2 and tetravalent LUAS-4 are able to cluster CLEC-2 on a cell surface and that the clustering is supported by Syk, presumably through the binding to two phosphorylated tails of the receptor.

### Tetravalent but not divalent ligands stimulate aggregation of human platelets

**Fig. 3:**
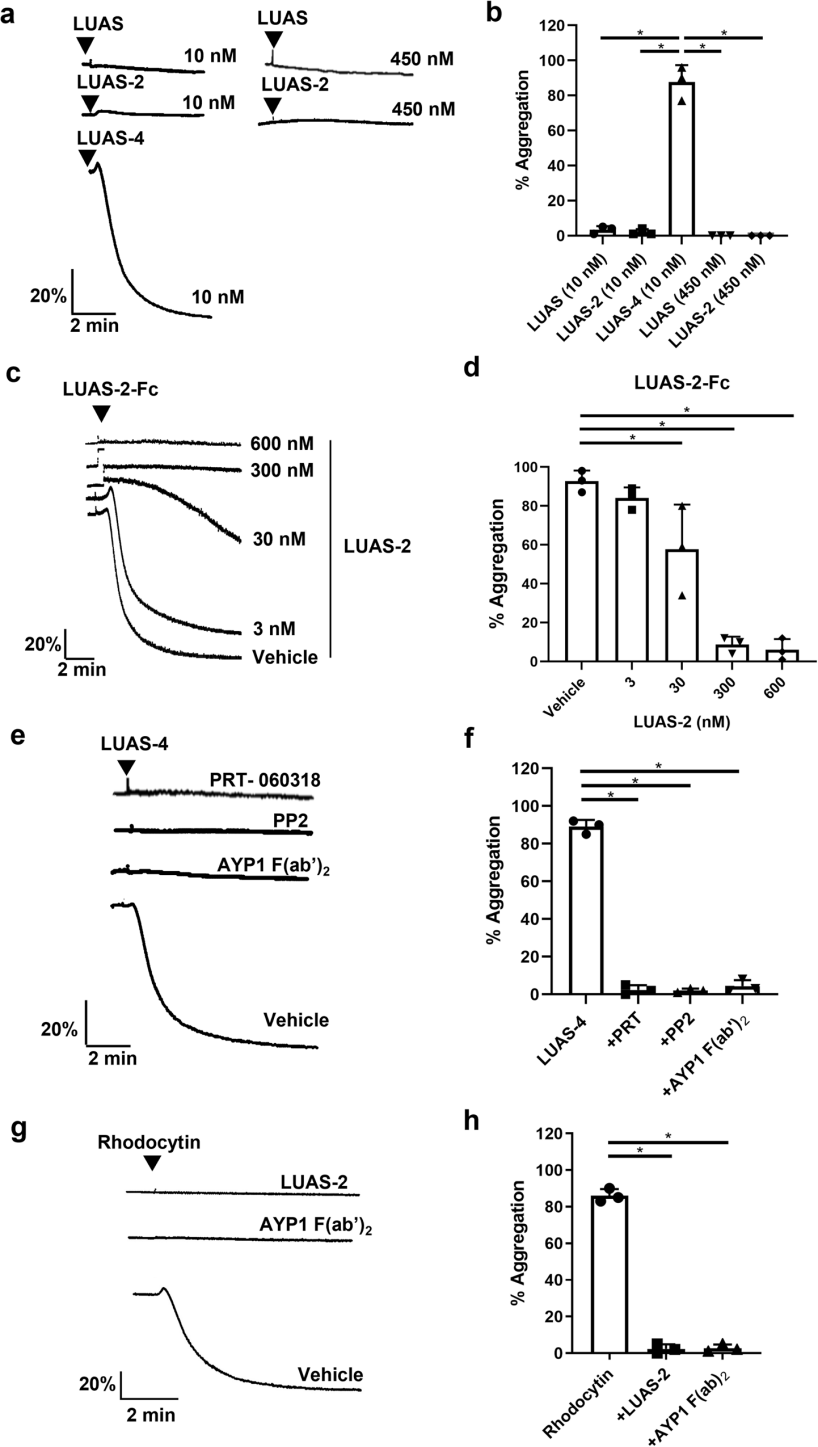
Human platelets are activated by tetravalent but not divalent CLEC-2 nanobodies. Platelet aggregation was monitored by light transmission aggregometry at 37 °C with constant stirring at 1200 rpm for 5–10 min. **a** Representative traces of platelet aggregation (2 × 10^8^ platelets/ml) following incubation with monovalent LUAS, divalent LUAS-2 and tetravalent LUAS-4 (10 nM). **b** Percentage of platelet aggregation induced by LUAS, LUAS-2 and LUAS-4 (*n* = 3 biologically independent experiments). **c** Representative traces of platelet aggregation induced by tetravalent LUAS-2-Fc (1.25 nM) in the absence (vehicle) and presence of increasing concentrations of divalent LUAS-2 (3, 30, 300, 600 nM). Platelets were preincubated for 5 min at 37 °C prior to stimulation. **d** Percentage of platelet aggregation induced by LUAS-2-Fc in the absence and presence of LUAS-2 (*n* = 3 biologically independent experiments). **e** Representative traces of platelet aggregation induced by LUAS-4 (10 nM) in absence (vehicle) and presence of PRT-060318 (1 μM), PP2 (20 μM) and AYP1 F(ab’)_2_ (66 nM). Platelets were preincubated with inhibitors for 5 min at 37 °C prior to stimulation. **f** Percentage of platelet aggregation induced by LUAS-4 with inhibitors (*n* = 3 biologically independent experiments). **g** Representative traces of platelet aggregation induced by rhodocytin (100 nM) in the absence (vehicle) and presence of LUAS-2 (10 nM), and AYP1 F(ab’)_2_ (66 nM). Platelets were preincubated with inhibitors for 5 min at 37 °C prior to stimulation. **h** Percentage of platelet aggregation induced by rhodocytin (100 nM) in the absence (vehicle) and presence of LUAS-2 (10 nM), and AYP1 F(ab’)_2_ (66 nM) (*n* = 3 biologically independent experiments). Significance was measured using one-way ANOVA with a Bonferroni post-hoc test where *P* ≤ 0.05. Data presented as mean ± SD.

As summarised in the introduction, divalent ligands such as mAbs and Fc-podoplanin have been shown to activate CLEC-2 in mouse platelets but not human platelets^8,21,27,28^. Several factors or a combination of factors could account for this including differences in receptor expression levels and both signalling and membrane-regulatory proteins in mouse and human platelets, and structural differences between the two receptors. To investigate this further, and to establish the relationship between ligand valency and activation of CLEC-2, we tested the ability of monovalent LUAS, divalent LUAS-2 and tetravalent LUAS-4 and LUAS-2-Fc nanobodies to induce aggregation of human platelets using light transmission aggregometry. LUAS and LUAS-2 did not cause aggregation of human platelets at 10 or 450 nM whereas LUAS-4 and LUAS-2-Fc induced rapid, sustained aggregation of platelets (Fig. 3a–d). Platelet aggregation induced by tetravalent LUAS-4 was blocked in the presence of AYP1 F(ab’)_2,_ and by inhibitors of Src and Syk kinases, confirming that activation is mediated through CLEC-2 (Fig. 3e,f, Supplementary Fig. 2a). Divalent LUAS-2 blocked platelet aggregation induced by tetravalent LUAS-2-Fc and the CLEC-2 snake venom toxin agonist rhodocytin (Fig. 3c, d, g, h). Together, the results suggest that tetravalent but not monovalent or divalent ligands are able to induce sufficient clustering of CLEC-2 to activate human platelets.

**Fig. 4:**
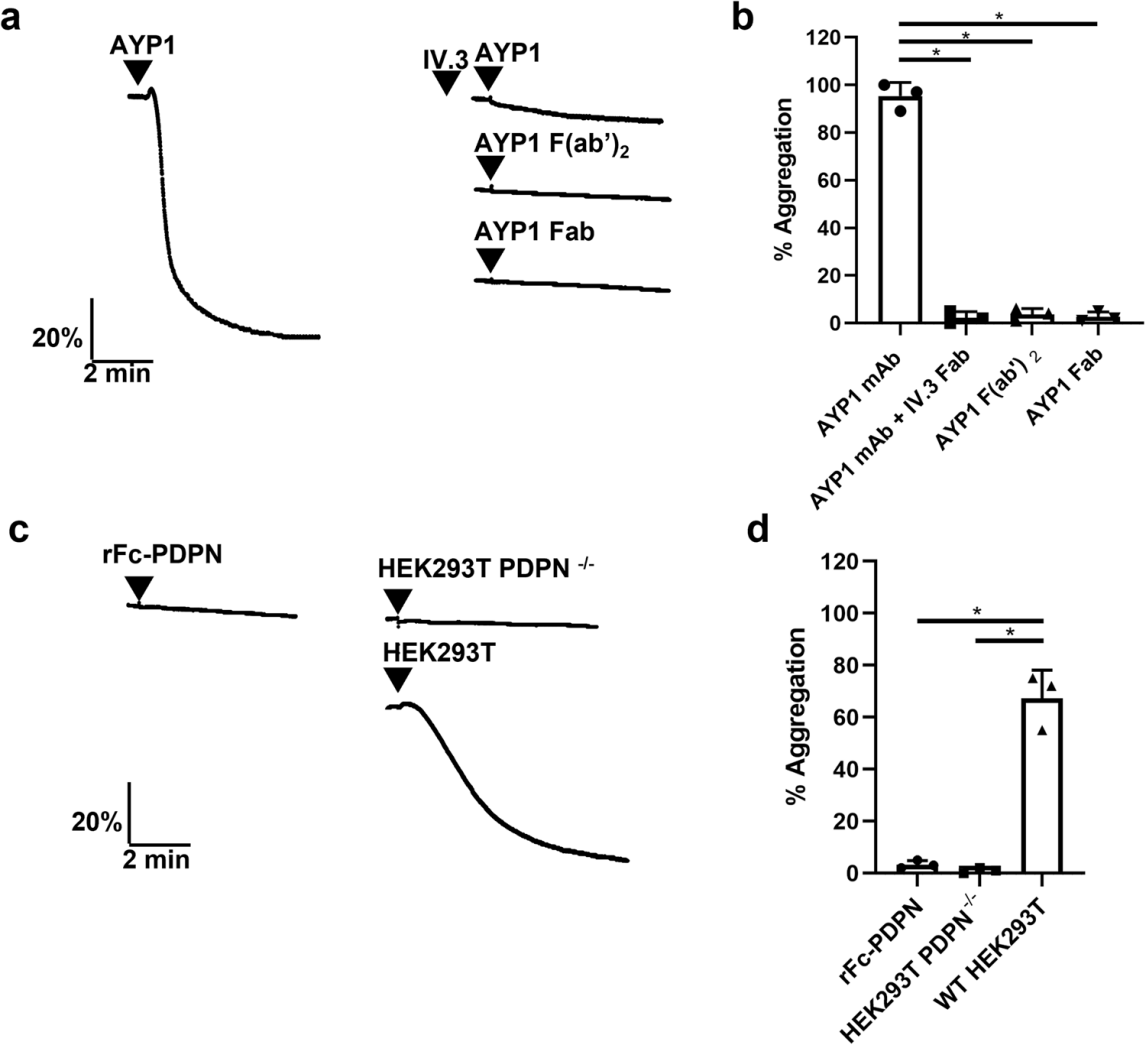
Divalent AYP1 and podoplanin do not activate human platelets. Platelet aggregation was monitored by light transmission aggregometry at 37 °C with constant stirring at 1200 rpm for 5 mins. **a** Representative traces of human platelet aggregation (2 × 10^8^ platelets/ml) induced by monoclonal antibody (mAb) AYP1 (66 nM) in the absence and presence of the anti-FcγRIIA IV.3 Fab (30 µg/ml), AYP1 F(ab’)_2_ (66 nM) and AYP1 Fab fragments (66 nM). Platelets were preincubated with IV.3 Fab for 10 min at 37 °C prior to stimulation. **b** Percentage of platelet aggregation induced by mAb AYP1 or AYP1 Fab fragments (*n* = 3 biologically independent experiments). **c** Representative traces of platelet aggregation following incubation with recombinant Fc-podoplanin (rFc-PDPN,136 nM), HEK293T PDPN^−/−^ cells (5 × 10^5^ cells) or wild-type HEK293T cells (5 × 10^5^ cells). **d** Percentage of platelet aggregation induced by rFc-PDPN, HEK293T PDPN^−/−^ cells or wild-type HEK293T cells (*n* = 3 biologically independent experiments). Significance was measured using one-way ANOVA with a Bonferroni post-hoc test where *P* ≤ 0.05. Data presented as mean ± SD.

To support this conclusion, we monitored aggregation of human washed platelets in response to the mAb AYP1 and its Fab fragments, podoplanin-expressing cells and recombinant Fc-podoplanin (rFc-PDPN). Full-length AYP1 stimulated aggregation of human platelets which was blocked by the Fab fragment of the anti-FcγRIIA mAb IV.3, demonstrating the dependency on the Fc domain of AYP1 (Fig. 4a, b). In contrast, neither AYP1 F(ab’)_2_ nor AYP1 Fab were able to induce aggregation (Fig. 4a, b). Podoplanin-expressing cells stimulated platelet aggregation whereas divalent rFc-PDPN had no effect (Fig. 4c, d). These results demonstrate that divalent CLEC-2 ligands do not cause aggregation of human platelets and act as antagonists, and that a minimum valency of greater than 2 is required to induce activation.

### Divalent ligands activate humanised CLEC-2 mouse platelets (*hCLEC1b*^*KI*^*)*

In contrast to human platelets, divalent ligands stimulate full aggregation of mouse platelets as illustrated by challenge with the mAb INU1 and rFc-podoplanin^8,21,27^ (mouse platelets do not express an Fc receptor and therefore are not affected by the presence of an Fc domain). These results indicate a species difference in the ability of divalent ligands to stimulate aggregation. To address whether this is due to a difference between mouse and human CLEC-2 or between mouse and human platelets, we used platelets from a humanised CLEC-2 knock-in transgenic mouse (*hCLEC1b*^*KI*^*)*^29^.

**Fig. 5:**
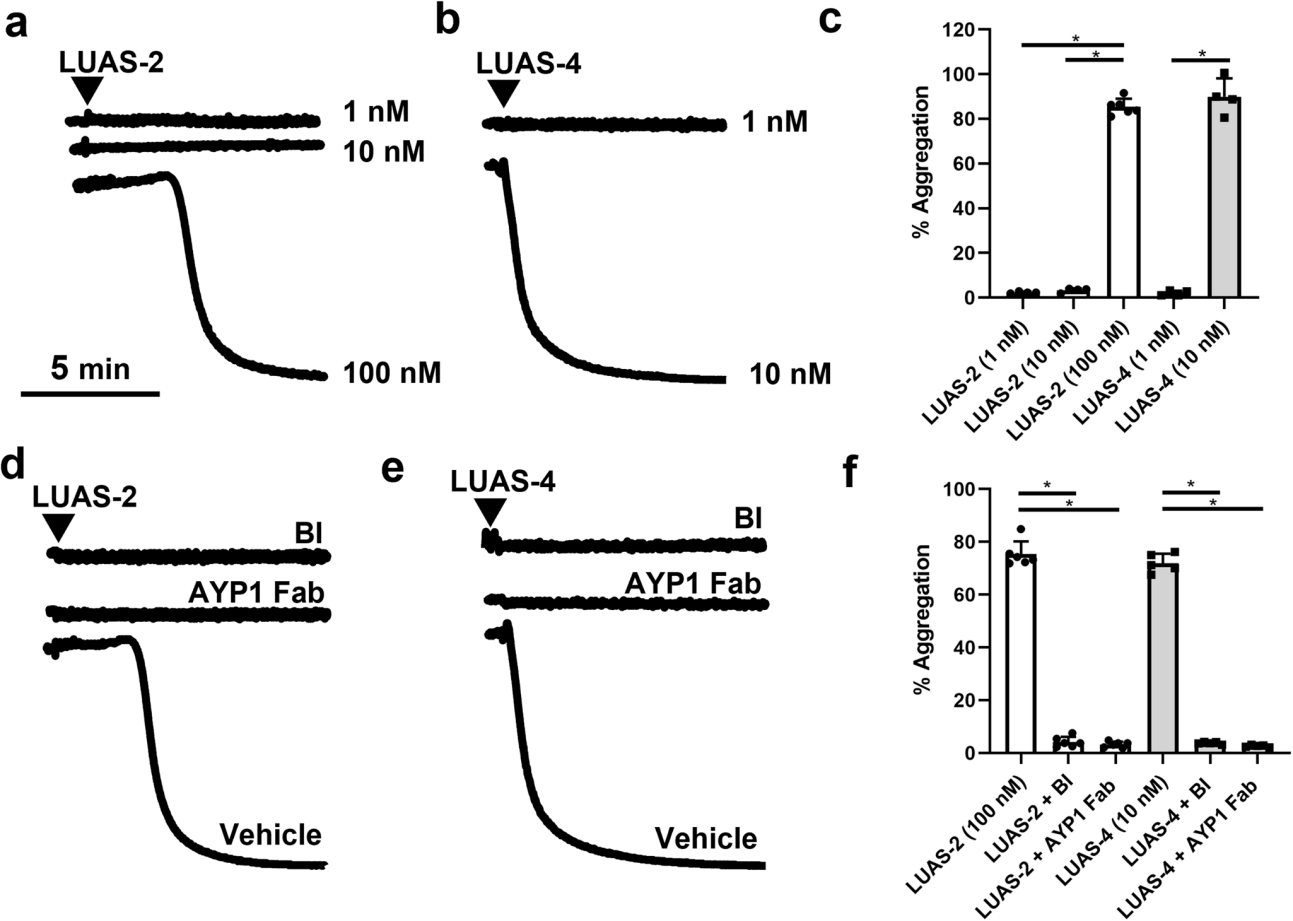
Divalent and tetravalent nanobodies activate human CLEC-2 in mouse platelets. Humanised CLEC-2 mouse (*hCLEC1b*^*KI*^) platelet aggregation was monitored by light transmission aggregometry at 37°C with constant stirring at 1200 rpm for 10 min. Representative traces of washed hCLEC-2^KI^ platelet aggregation (2 × 10^8^ platelets/ml) induced by (**a**) LUAS-2 (1-100 nM) and (**b**) LUAS-4 (1-10 nM). **c** Percentage of maximal platelet aggregation induced by LUAS-2 and LUAS-4 (*n* = 4–6 biologically independent experiments). Representative traces of washed hCLEC-2^KI^ platelet aggregation (2 × 10^8^ platelets/ml) induced by (**d**) LUAS-2 (100 nM) and (**e**) LUAS-4 (10 nM) in the absence (vehicle) or presence of Syk inhibitor BI1002494 (BI) (500 nM) and AYP1 Fab (208 nM). Platelets were preincubated with inhibitors for 5 min at 37 °C prior to stimulation. **f** Percentage of platelet maximal aggregation induced by LUAS-2 and LUAS-4 in the presence of BI or AYP1 Fab (*n* = 5–6 biologically independent experiments). Significance was measured with a one-way ANOVA with Bonferroni *post-hoc* for where *P* ≤ 0.05. Data are presented as mean ± SD.

In contrast to the results in human platelets, the divalent nanobody LUAS-2 stimulated rapid and full aggregation of *hCLEC1b*^*KI*^ mouse platelets (Fig. 5a–c). LUAS-4 also stimulated aggregation but at a 10x lower concentration than LUAS-2 due to the increase in avidity (Fig. 5b, c). Aggregation induced by LUAS-2 and LUAS-4 was blocked by AYP1 Fab and the Syk inhibitor, BI1002494^30^, confirming that aggregation is mediated through the activation of CLEC-2 (Fig. 5d–f, Supplementary Fig. 2b, c).

These data demonstrate that a divalent ligand to CLEC-2 is an antagonist in human platelets but an agonist in mouse platelets expressing the human CLEC-2 receptor. A similar result is also seen with the mAb AYP1 which recognises human CLEC-2^29^. Thus, this differential action is not due to a difference between human and mouse CLEC-2, indicating an inherent difference between human and mouse.

### Divalent ligands activate CLEC-2 in high but not low-expressing DT40 cells

**Fig. 6:**
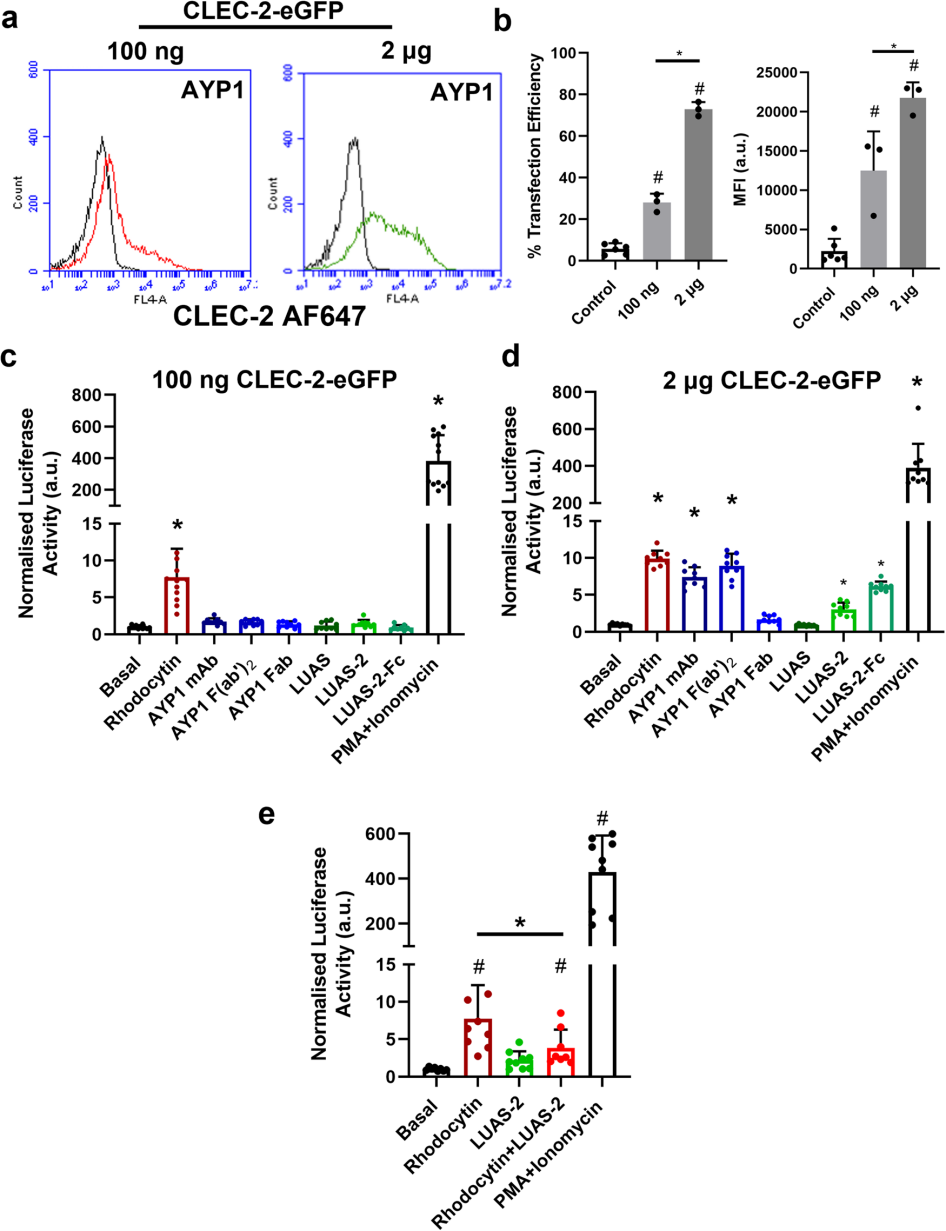
Divalent and tetravalent ligands activate CLEC-2 in high- but not low-expressing transfected cells. **a** Expression of CLEC-2-eGFP (100 ng and 2 μg) in DT40 chicken B cells was measured by flow cytometry using the anti-CLEC-2 AYP1 antibody (66 nM) with anti-mouse Alexa Fluor-647 secondary staining. The NFAT-luciferase reporter was also co-transfected. Black histograms show non-specific secondary staining alone. **b** Flow cytometry data presented as the transfection efficiency and MFI of CLEC-2-eGFP construct (100 ng and 2 μg) showing percentage of cells positive (*n* = 3 biologically independent experiments). **c**, **d** DT40 cells were transfected with an NFAT-luciferase reporter construct, and either 100 ng or 2 μg of the CLEC-2-eGFP construct. Cells were unstimulated or stimulated for 6 h and then lysed and assayed for luciferase activity. Luciferase activity normalised for basal values for 100 ng and 2 μg CLEC-2-eGFP unstimulated and stimulated with rhodocytin (30 nM), AYP1 mAb (6.6 nM), AYP1 F(ab’)_2_ (9.1 nM) and Fab fragments (20.8 nM), LUAS (10 nM), LUAS-2 (10 nM), LUAS-2-Fc (10 nM) and PMA (50 ng/ml) + ionomycin (1 μM) (positive control). Each experiment was performed in triplicate. **e** LUAS-2 is an antagonist to rhodocytin. Cells were unstimulated or stimulated for 6 h. Luciferase activity normalised for basal values for CLEC-2-eGFP (100 ng) unstimulated and stimulated with rhodocytin (30 nM), LUAS-2 (3 μM), rhodocytin (30 nM) + LUAS-2 (3 μM) and PMA (50 ng/ml) + ionomycin (1 μM) (positive control). Cells were incubated with LUAS-2 for 30 min prior to rhodocytin addition. In (**b**) and (**e**) # = statistical significance compared to control/basal. In (**c**) and (**d**) * = statistical significance compared to basal. Significance was measured with either a one-way ANOVA with Bonferroni post-hoc for (**b**) and Student two-tailed *t* test for (**c**–**e**) where *P* ≤ 0.05. All data are presented as mean ± SD (*n* = 3 biologically independent experiments).

Mouse platelets have been reported to have 10–20 times higher level of expression of CLEC-2 than human platelets using proteomic and antibody-bead labelling methodology^31–33^. Consistent with this, the *hCLEC1b*^*KI*^ mouse platelets have a greater level of expression of CLEC-2 than human platelets (Supplementary Fig. 3a)^29^. The higher expression of CLEC-2 in mouse relative to human platelets may have contributed to the ability of divalent ligands to serve as agonists. To investigate this, we used a transfected DT40 B cell model with a nuclear factor of activated T cells (NFAT) reporter expressing different levels of CLEC-2-eGFP (Fig. 6a, b and Supplementary Fig. 3a). The human CLEC-2-transfected DT40 cells support signalling by the snake venom toxin rhodocytin^34,35^, a result that has been confirmed in Fig. 6. Strikingly, however, neither divalent AYP1 and LUAS-2 nor tetravalent LUAS-2-Fc stimulated NFAT activation in the low-expressing CLEC-2 transfected cell line model (Fig. 6c). The combination of PMA and ionomycin which stimulate MAPK signalling via PKC activation and Ca^2+^ release respectively, gave robust activation of the NFAT reporter (Fig. 6c).

In contrast to the low-expressing cells, divalent AYP1 mAb and AYP1 F(ab’)_2_, and tetravalent LUAS-2-Fc stimulated a similar increase in NFAT activity to that induced by rhodocytin, while the divalent nanobody LUAS-2 stimulated ~50% of the response in the high-expressing CLEC-2-eGFP cells (Fig. 6d). As expected, monovalent AYP1 Fab fragments and LUAS did not stimulate NFAT activity. These results show that a divalent and tetravalent but not a monovalent ligand is able to activate human CLEC-2 in high- but not low-expressing transfected DT40 cells.

We next investigated whether LUAS-2 could inhibit the NFAT response following rhodocytin stimulation of CLEC-2 in the low-expressing cells where LUAS-2 does not induce receptor activation. LUAS-2 blocked the response to rhodocytin by ~50% (Fig. 6e), thereby showing that it is an antagonist in low-expressing CLEC-2 cells. This is in agreement with the results seen in human platelets.

### Single-molecule microscopy shows CLEC-2 is a mixture of monomers and dimers in the membrane

CLEC-2 has been shown to form dimers in the membrane as reviewed by Martin et al.^11^ but the stoichiometry of dimerisation in the membrane and how this is governed by the level of expression is not known.

**Fig. 7:**
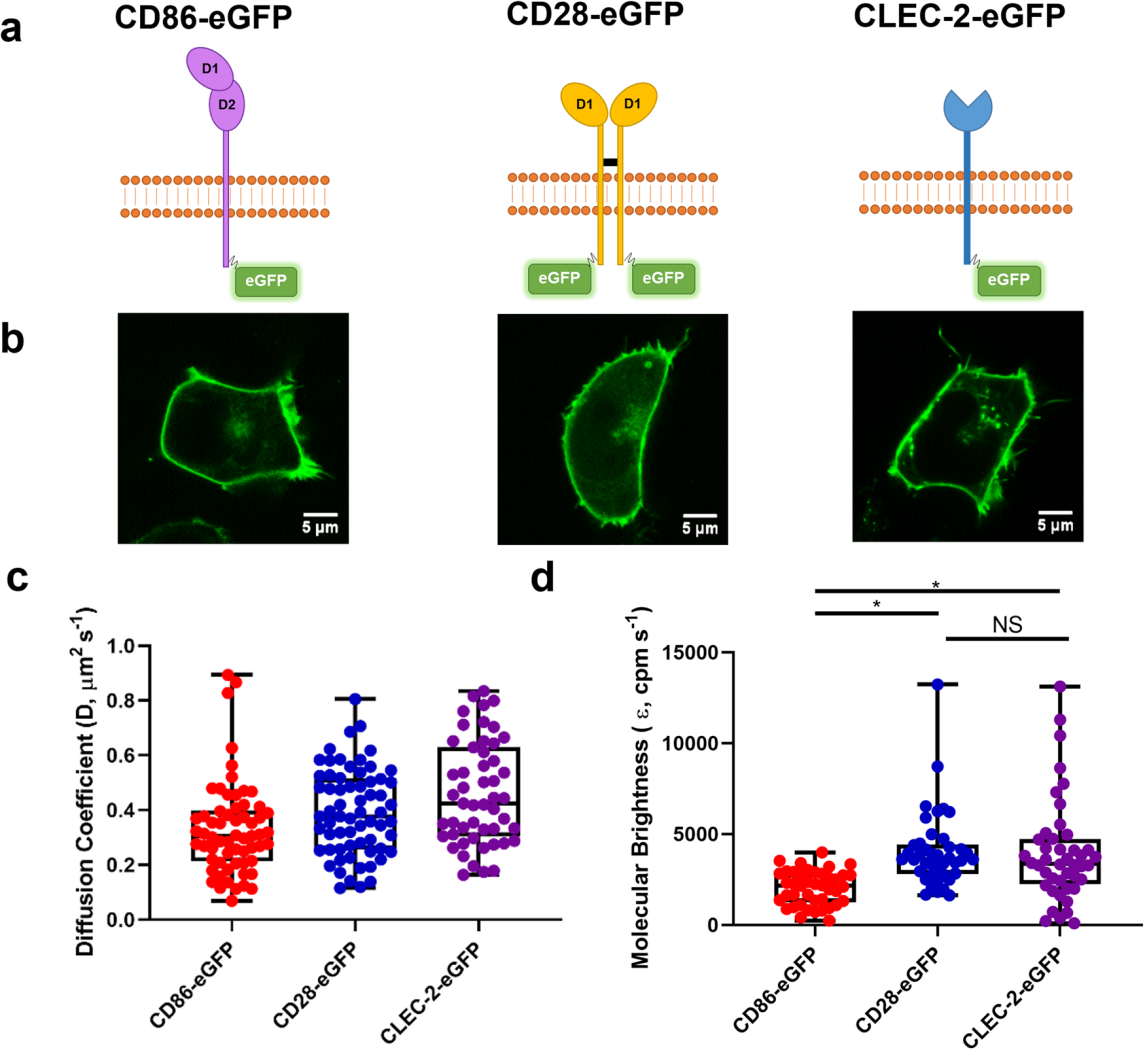
Fluorescence correlation spectroscopy (FCS) shows CLEC-2 is a mixture of monomers and dimers. **a** Schematic representation of C-terminal CD86-eGFP monomer control, CD28-eGFP dimer control and N-terminal human CLEC-2-eGFP. eGFP tags have an A206K mutation to prevent eGFP dimerisation. D1 = domain 1 and D2 = domain 2. **b** Representative confocal microscopy images showing membrane localisation of CD86-eGFP, CD28-eGFP and CLEC-2-eGFP in transfected HEK293T cells (field of view = 52 × 52 μm) (scale bar = 5 μm). **c** Box plot of CD86, CD28, and CLEC-2 diffusion coefficient data in HEK293T cells. The diffusion coefficients were calculated from the derived autocorrelation fits. **d** Photon counting histogram (PCH) analysis of CD86, CD28 and CLEC-2 to determine molecular brightness (*ε*, counts per molecule per second, cpm s^−1^) and stoichiometry of the receptors in HEK293T cells. 1-component PCH fitting was applied to raw PCH. NS = not significant. For all box plots, centre lines represent the median; box limits indicate the 25th and 75th percentiles and whiskers extend to minimum and maximum points. Significance was measured with Kruskal-Wallis with Dunn’s post-hoc where *P* ≤ 0.05. FCS measurements were taken in 53–70 cells (*n* = 6 biologically independent experiments).

To address this, we have used FCS and single molecule stepwise photobleaching in transfected HEK293T cells expressing the N-terminal eGFP-tagged CLEC-2. The results have been compared to eGFP-tagged versions of the monomeric receptor CD86 and the dimeric receptor CD28 (Fig. 7a, b). Measurement of time-dependent fluorescence intensity fluctuations (Supplementary Fig. 1b) generated autocorrelation curves and PCHs for determination of diffusion coefficients and molecular brightness, respectively (Supplementary Fig. 1c, d). FCS measurements of CLEC-2, CD86 or CD28 expressing cells showed similar diffusion coefficients of 0.46 ± 0.18, 0.33 ± 0.17 and 0.39 ± 0.16 μm^2^ s^−1^ respectively, as expected due to their similar size (Fig. 7c). PCH analysis was applied to the FCS measurements to calculate the molecular brightness which is proportional to the number of eGFP molecules within a fluorescent protein complex. As expected, the molecular brightness measurements of CD86-eGFP and CD28-eGFP differed by ~2-fold due to their monomeric and dimeric stoichiometry (Fig. 7d). The molecular brightness of CLEC-2 was intermediate to that of CD86 and CD28 indicating that CLEC-2 is expressed as a mixture of monomers and dimers in the membrane (Fig. 7d).

**Fig. 8:**
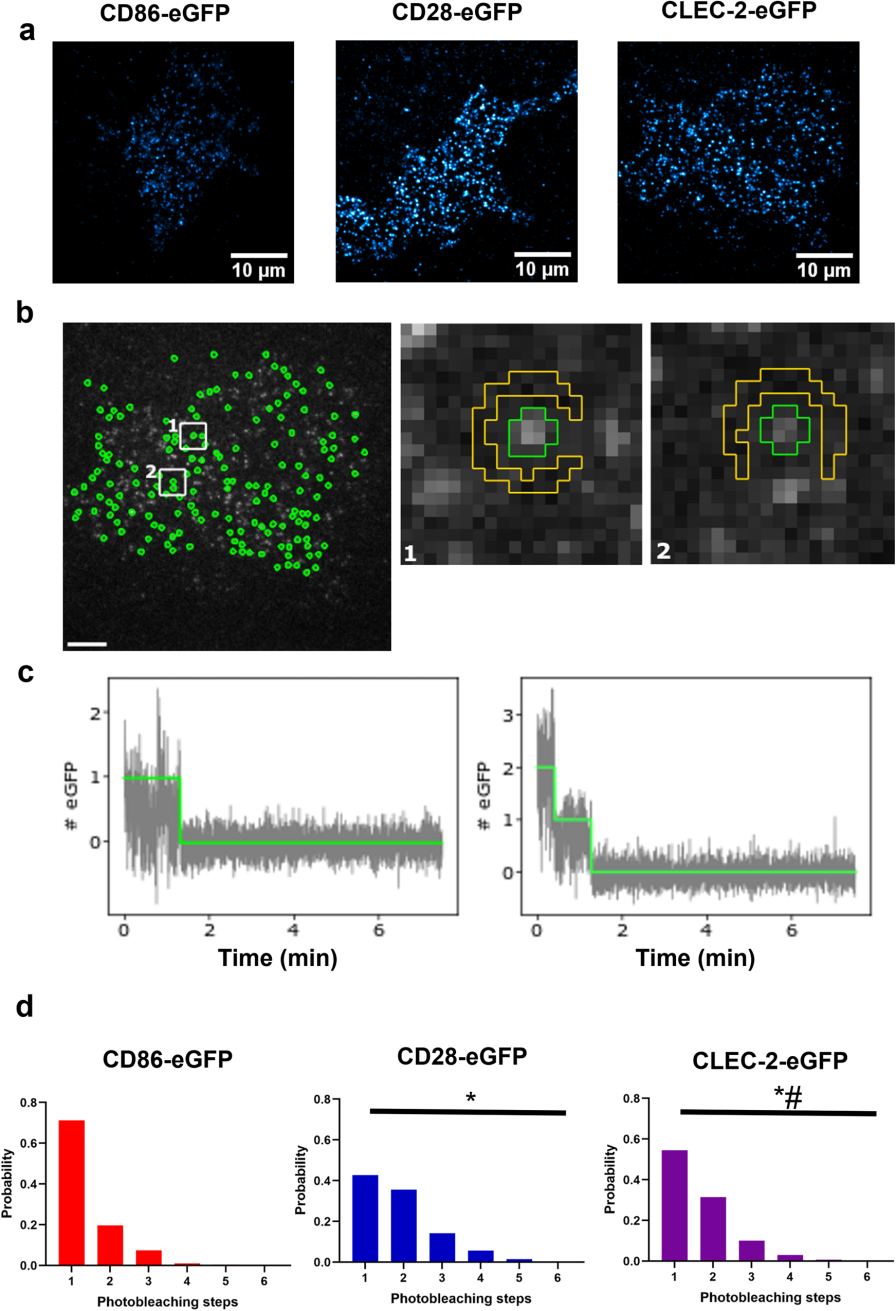
Single-molecule stepwise photobleaching analysis shows CLEC-2 is a mixture of monomers and dimers. **a** Representative total internal reflection fluorescence (TIRF) microscopy images with average intensity projections of the first 100 frames of the basal plasma membrane of transfected HEK293T cells expressing CD86-eGFP, CD28-eGFP and human CLEC-2-eGFP showing individual fluorescent spots visualised with lookup table cyan hot (field of view = 40 × 40 μm) (scale bar = 10 μm). **b** Representative image of the fluorescent spot detection following application of an automated spot detection algorithm of the entire basal membrane of the cell expressing CLEC-2-eGFP. Detected spots included in the analysis are shown in green and detected background is shown in yellow in panels 1 and 2 (scale bar = 5 μm). **c** Fluorescence intensity decay traces (grey) for individual spots showing photobleaching steps determined by the algorithm (green line). **d** Photobleaching step frequency histograms determined from all accepted spots for CD86-eGFP (red), CD28-eGFP (blue) and CLEC-2-eGFP (purple) in HEK293T cells. Significance of the distributions was measured with Epps-Singleton 2 sample test where *P* ≤ 0.05. * = significance compared to CD86-eGFP and # = significance compared to CD28-eGFP. Data pooled from 355 to 1008 traces (*n* = 3 biologically independent samples.

Stepwise photobleaching studies on HEK293T cells expressing low levels of eGFP-tagged CLEC-2, CD86 or CD28 were carried out to further investigate the membrane organisation of CLEC-2. The density of CLEC-2, CD86 and CD28 receptors in this study was similar with 0.60 + 0.26, 0.56 + 0.22 and 0.68 + 0.25 localisations/μm^2^, respectively (Supplementary Fig. 4a–c). This experiment is challenging to interpret in cells with a higher level of expression due to the co-localisation of complexes as a result of the density. The percentage of undetectable eGFP molecules (due to maturation issues or premature photobleaching) and overlapping spots were estimated to be ~40% and ~28%, respectively (Supplementary Fig. 4d, e). The number of receptors in a fluorescent detection (spot) is determined by photobleaching. Spots containing an eGFP-tagged monomer decay in one bleach step, whereas spots containing dimers or oligomers decay in multiple discrete steps, with the number of steps reflecting the stoichiometry of the protein complex. Distinct fluorescent protein spots of the three receptors were visualised by total internal reflection fluorescence (TIRF) microscopy (Fig. 8a) and were photobleached over time. Fluorescence intensity traces associated with individual spots isolated from background were extracted and the number of photobleaching steps in each trace was determined (Fig. 8b, c)^36^. Photobleaching step histograms show that majority (~71% of spots) of CD86-eGFP spots bleached in a single step consistent with a monomeric stoichiometry (Fig. 8d). The fraction (~29%) of traces requiring more than one step could reflect the presence of coincidently overlapping spots. In contrast, CD28-eGFP contained a larger proportion of spots that bleached in two or more steps (~57% of spots) consistent with expression as a covalent-linked dimer (Fig. 8d). In agreement with the FCS studies, the photobleaching step histogram of CLEC-2 lies in between that of CD86 and CD28 indicating that CLEC-2 is expressed as a mixture of monomers and dimers (Fig. 8d). We estimate that ~60% of CLEC-2 fluorescent spots are present as a homodimer at this level of expression (Supplementary Fig. 4f).

The photobleaching studies are therefore in agreement with the FCS measurements that CLEC-2 is expressed as a mixture of monomers and dimers in the membrane.

### Dimerisation of CLEC-2 increases with expression

We have shown above that receptor expression is an important variable that influences the agonist or antagonist behaviour of divalent ligands. While this could be explained by an increase in receptor density, it could also reflect an increase in receptor dimerisation with increasing expression, as a result of increased receptor collisions in the membrane. This would enable a divalent ligand to crosslink an increased proportion of dimeric receptors to each other or to a monomeric receptor at the expense of crosslinking two monomeric receptors.

**Fig. 9:**
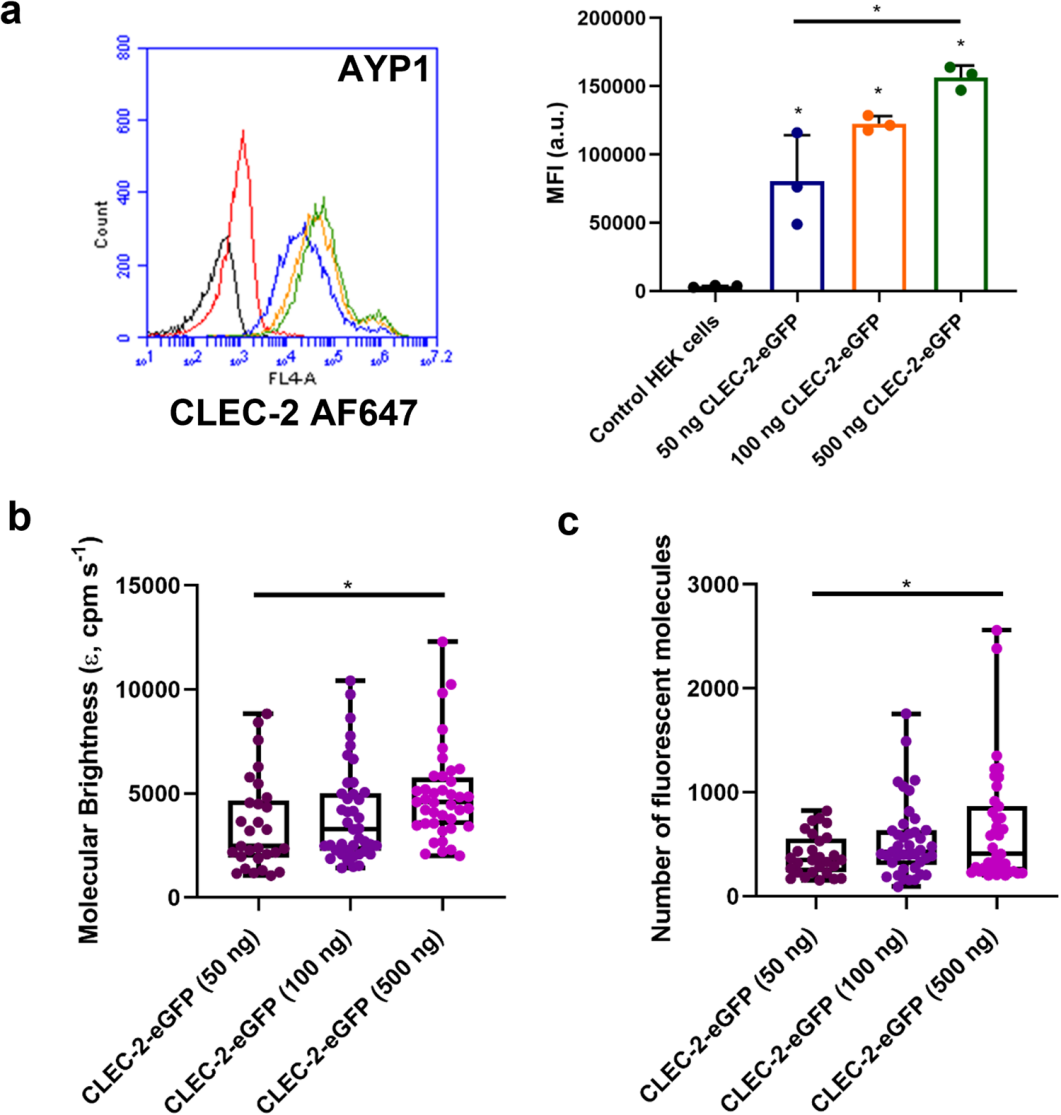
CLEC-2 dimerisation increases with receptor expression. **a** Wild-type HEK293T cells were transfected with 50, 100 or 500 ng of CLEC-2-eGFP DNA by PEI. CLEC-2-eGFP expression in HEK293T cells was measured by flow cytometry using mAb AYP1 (10 µg/ml) with Alexa Fluor-647 anti-mouse secondary antibody. Representative fluorescence intensity histograms for 50 (Blue), 100 (Orange) and 500 (Green) ng CLEC-2-eGFP transfected HEK293T samples. The black histogram shows HEK293T cells with no stain and the red histogram shows non-specific secondary staining alone. Flow cytometry data presented as MFI (a.u.). Data presented as mean ± SD (*n* = 3 biologically independent experiments). Box plots showing the effect of increasing receptor expression on CLEC-2-eGFP (**b**) molecular brightness (ε, counts per molecule per second, cpm s^−1^) and (**c**) number of fluorescent molecules within the confocal volume measured by FCS. For all box plots, centre lines represent the median; box limits indicate the 25th and 75th percentiles and whiskers extend to minimum and maximum points. Significance was measured with a one-way ANOVA with Bonferroni post-hoc for (**a**) and a Kruskal-Wallis with Dunn’s post-hoc for (**b**–**c**) where *P* ≤ 0.05. FCS measurements were taken in 45–67 cells (*n* = 3 biologically independent experiments).

To investigate the relationship between receptor expression and dimerisation, we performed FCS on HEK293T cells transfected with increasing levels of CLEC-2-eGFP. Flow cytometry was used to show that the expression of CLEC-2 increases with the amount of DNA transfected (Fig. 9a). In line with this, the molecular brightness of CLEC-2 increases with the amount of DNA (Fig. 9b, c), with a 2.3-fold greater level of brightness compared to monomeric CD86 at the highest level of CLEC-2 expression. This shows that there is increased dimerisation and higher-order clustering of CLEC-2 as the receptor density increases, and that receptor dimerisation may therefore be an important mediator in the agonist or antagonist activity of divalent ligands.

### CLEC-2 is a mixture of monomers and dimers in human platelets

The above methods cannot be applied to study the stoichiometry of CLEC-2 in platelets as they cannot be transfected. For this reason, we have used styrene maleic acid lipid particles (SMALP) technology in combination with native PAGE to determine the configuration of CLEC-2 in resting platelets. This approach overcomes the artificial clustering of proteins in the membrane induced by detergents due to loss of membrane lipids. Styrene maleic acid forms ~10 nm diameter discs in the membrane known as SMALPs that preserve membrane proteins and their surrounding lipids^37,38^. The intact SMALPs containing the protein of interest can be resolved by native gel electrophoresis (SMA-PAGE)^39^, thus preserving protein complexes, and the protein content identified by western blotting. The recombinant C-type lectin-like domain of CLEC-2, which is monomeric^13^, was used to study the migration of CLEC-2 on native SMA-PAGE as the migration of proteins cannot be accurately predicted by their molecular weight.

**Fig. 10:**
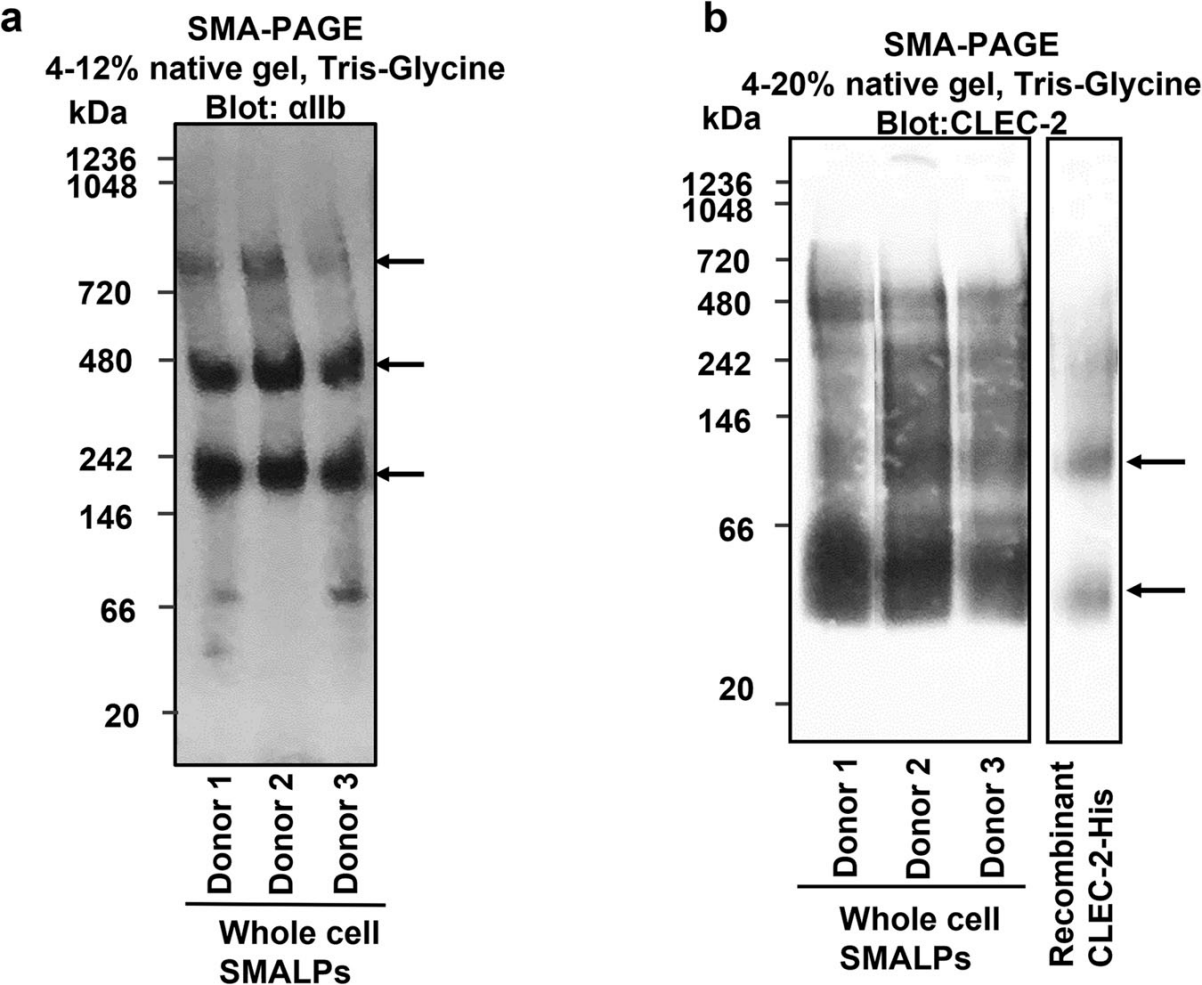
αIIbβ3 and CLEC-2 blots on native PAGE with human platelet SMALP samples. Resting-state platelets from three donors were isolated and whole cell SMALPs were generated as described in methods. Platelet SMALPs were then subjected to native-PAGE (SMA-PAGE), where the negatively charged SMA belt of the nanodisc provides the migration-driven electronic force, with the gel percentage indicated. After electrophoresis, the proteins were transferred onto PVDF membrane and blotted with anti-αIIb (**a**) or anti-CLEC-2 AYP2 (**b**) antibodies. **a** αIIb blot on platelet SMALPs from three donors shows the existence of αIIb heterodimer and higher order oligomers. **b** Platelet SMALPs from three donors showed similar band patterns with a major band for platelet CLEC-2 just above the recombinant C-type lectin-like domain (Recombinant CLEC-2-His) control and a minor band for both proteins at twice the size (*n* = 3 biologically independent samples).

We first measured the migration of integrin αIIbβ3, following SMALPing on native PAGE to demonstrate resolution of a protein dimer. As shown in Fig. 10a, αIIbβ3 runs at the expected molecular size for a heterodimer (~245 kDa^40^), with a second minor band at twice this molecular weight suggesting that it is also present as a dimer of heterodimers. This has not previously been described in platelets, although clusters of the integrin have been shown by expansion microscopy^41^. CLEC-2 has a predicted molecular weight of ~40 kDa taking into account glycosylation^11^. Fig. 10b shows that the major band for CLEC-2 migrated just above the slightly smaller monomeric recombinant C-type lectin-like domain control with a minor band for both proteins migrating at twice the size. This indicates that CLEC-2 is a mixture of monomers and dimers in human platelets, with monomers predominating over dimers. This result is in line with the studies using FCS and single molecule photobleaching in transfected HEK293T cells described above, and previous crosslinking studies in human platelets^12^.

## Discussion

In the present study, we show that (i) a novel divalent nanobody to CLEC-2, LUAS-2, functions as an antagonist to the snake venom toxin rhodocytin in human platelets and low-expressing CLEC-2 DT40 cells, and as an agonist in humanised CLEC-2 mouse platelets (which express a higher level of CLEC-2) and high-expressing DT40 cells; (ii) a tetravalent nanobody, LUAS-4 or LUAS-2-Fc, functions as an agonist in human platelets showing that more than two epitopes are required for activation of human platelets; (iii) CLEC-2 is expressed as a mixture of monomers and dimers in transfected cell lines and platelets and that increasing expression of CLEC-2 drives dimerisation; (iv) divalent and tetravalent ligands but not monovalent ligands drive dimerisation/oligomerisation of CLEC-2; (v) dimerisation/oligomerisation of CLEC-2 is regulated by the tyrosine kinase Syk. These results demonstrate that ligand valency, receptor density and Syk regulate receptor clustering and show that divalent ligands can act as antagonists or agonists depending in part on the receptor density. Not only will an increase in receptor density drive a bigger signal, but it will also increase the proportion of receptor dimers enabling divalent ligands to crosslink dimers and form larger clusters of CLEC-2.

The antagonist and agonist activity of the divalent nanobody in human platelets and humanised CLEC-2 mouse platelets, respectively, is also seen with a second divalent ligand, the F(ab’)_2_ fragment of the mAb to human CLEC-2, AYP1^29^. Further, a mAb against mouse CLEC-2, INU1, which is also divalent, is an agonist in wild-type mouse platelets^21,22^. The results show that the agonist and antagonist activity of divalent ligands is not unique to LUAS-2 or to human CLEC-2.

The advanced microscopy techniques of FCS and single molecule photobleaching were used to show that CLEC-2 is expressed as a mixture of monomers and dimers in transfected cell lines, and that the degree of dimerisation increases with receptor density. The results are in line with those using a non-detergent method of extraction in resting platelets (SMALP technology) which reveal that CLEC-2 is a mixture of monomers and dimers. The results provide a semi-quantitative measurement of the degree of dimerisation of CLEC-2 in a resting cell and show that the level of dimerisation in the membrane increases upon expression. This is consistent with SPR measurements showing that recombinant human and mouse CLEC-2 extracellular regions undergo homodimerisation with an affinity in the order of 300–500 nM^15^. Thus, while CLEC-2 is expressed in the membrane as a monomer, it is able to undergo dimerisation on collision with another CLEC-2, leading to a mixture of monomers and dimers.

The agonist and antagonist activity of the divalent ligands of CLEC-2 is reminiscent of that of partial agonists of G protein-coupled receptor (GPCR) ligands which can also serve as agonists or antagonists. This difference in activity is known as intrinsic activity and is encompassed in the term efficacy^42,43^. The ability of partial agonists of GPCRs to serve as agonists or antagonists is determined by receptor density and the level of signalling and other regulatory proteins^44^. This is also the case with CLEC-2, with the degree of clustering determined by the receptor density and the level of Syk, and the resulting intracellular signal is also governed by the level of other regulatory proteins such as tyrosine phosphatases.

CLEC-2 surface levels on human platelets have been reported to be ~2000–4000 copies per platelet^20,31,33^ but to be elevated in severely obese patients along with upregulation of phosphoproteins involved in Src kinase signalling pathway^45^. Elevated levels of soluble CLEC-2 have also been reported in patients with cardiovascular diseases and stroke although this could be due to increased shedding rather than expression^46–49^. It is possible that the increase in expression of CLEC-2 in obese and other patient groups has contributed to the increase in activity of circulating platelets as a result of increased clustering of the receptor.

The present study reports that two related tetravalent ligands, LUAS-2-Fc and LUAS-4, are powerful agonists on human platelets demonstrating that tetramerisation of CLEC-2 is sufficient to drive activation. An advantage of the LUAS-2-Fc construct was an increase in both yield and stability relative to LUAS-4. It should be noted that the Fc domain in LUAS-2-Fc is of mouse origin and does not bind to the low-affinity Fc receptor on human platelets, FcγRIIA. We observed activation of NFAT by rhodocytin but not by the tetravalent nanobody ligand, LUAS-2-Fc in low-expressing CLEC-2 transfected cells possibly reflecting the higher valency (octavalent)^50^ of the snake venom toxin. This further shows the importance of ligand valency in determining agonist activity of low-expressing cells.

It is of interest to consider whether this difference in reactivity to divalent ligands between mouse and human platelets also applies to other ITAM receptors. Human platelets express two ITAM receptors, FcγRIIA and GPVI, and mouse platelets express GPVI. The F(ab’)_2_ fragment of the mAb to FcγRIIA is widely used to block the Fc receptor in human platelets and has not been reported to induce activation. The mAb JAQ1 which binds to human and mouse GPVI is unable to activate human platelets on its own^51^ but potentiates activation of mouse platelets^52^. We speculate that the presence of FcγRIIA on human platelets may be one reason why they are less sensitive than mouse platelets to divalent ligands, as this will counter activation by circulating IgG and small immune complexes in the blood. The relative insensitivity of human platelets to divalent ligands may therefore be part of an evolutionary mechanism to limit the activation of ITAM receptors. Further, we have proposed that CLEC-2 and GPVI function as pattern recognition receptors for a miscellaneous group of charged ligands which includes circulating hormones such as adiponectin^53^. The relative insensitivity of human platelets to divalent ligands will also reduce the incidence of activation by this group of stimuli.

The observation that a tetravalent ligand induces powerful activation of CLEC-2 has clinical relevance in heparin-induced-thrombocytopenia (HIT) and vaccine-induced immune thrombocytopenia with thrombosis (VITT). Both conditions are caused by the generation of high-affinity antibodies to platelet factor 4 (PF4) which is tetravalent. The tetrameric structure favours the formation of large clusters of IgG as illustrated in Supplementary Fig. 5. The higher affinity of VITT antibodies for PF4 than HIT antibodies will favour the formation of larger clusters of FcγRIIA on the platelet surface and may explain the independence from heparin^54,55^.

In conclusion, we show that CLEC-2 is expressed as a mixture of monomers and dimers in platelets and cell lines, and that divalent ligands to CLEC-2 can function as agonists or antagonists with receptor number being one of the variables in determining their agonist or antagonist behaviour. This differential response to divalent ligands between human and mouse platelets may have important implications for studies focusing on mouse platelet biology. It may therefore be appropriate to consider divalent ligands to hemITAM receptors as partial agonists^56^.

The study has therefore advanced our understanding of the regulation of CLEC-2 clustering and signalling which will aid the development of therapeutics that are effective against clustering under all circumstances. It is likely that a monovalent CLEC-2 ligand will be most suitable for development in the clinic as a monovalent ligand always has an antagonistic action. While a divalent ligand has a greater affinity and, in the donors tested in this study, does not cause activation, we cannot rule out that it may cause activation in donors that express supramaximal levels of CLEC-2 or have an altered level in other CLEC-2 regulatory proteins.

## Methods

### Materials

Rhodocytin was purified from the venom of *Calloselasma rhodostoma* as described^57^. PEI (PEI Max MW 40,000) was purchased from Polysciences (Pennsylvania, USA). Enhanced chemiluminescence substrate (ECL) was obtained from ThermoFisher. The Src-family kinases inhibitor PP2 and Syk inhibitor PRT-060318 were purchased from Tocris (Abingdon, UK) and Caltag Medsystems (Buckingham, UK), respectively. The Syk inhibitor BI1002494^30^ was provided by Boehringer Ingelheim (Berkshire, UK). Anti-6-His IgG Alexa Fluor 647 and Anti-mouse IgG Alexa Fluor 647 were from Invitrogen, ThermoFisher Scientific (Paisley, UK). Other reagents were obtained from Merck Life Science UK Limited. Information about constructs can be found in the Supplementary Methods Section.

### Generation of multivalent LUAS nanobodies

The LUAS-2 and LUAS-4 constructs were created using a short flexible (G_4_S)_3_ linker between two or four copies of the original LUAS nanobody protein sequence respectively. Expression and purification of LUAS, LUAS-2 and LUAS-4 from E. coli WK6 cells is as reported^58^ (for the monovalent GPVI Nb2) and detailed in the Supplementary Methods Section. In short, E. coli WK6 cells were used to express the Nb constructs which contain an N-terminal PelB leader sequence to secrete protein into the periplasmic space as well as a C-terminal His-6 tag used for purification. An additional tetravalent construct, LUAS-2-Fc, was generated by addition of a mouse Fc domain (IgG2a) at the C-terminus of LUAS-2. This construct provided higher protein yield compared to LUAS-4 by utilising expression in mammalian cells, as described in detail in the Supplementary Methods Section. Purification of LUAS, LUAS-2, LUAS-4 and LUAS-2-Fc was achieved using two-step column chromatography (IMAC followed by size exclusion) and purity confirmed by SDS-PAGE.

### Antibodies

The generation of the CLEC-2 monoclonal antibody AYP1 and AYP2 has been reported^20^. AYP1 was produced in house using the generated hybridoma cell lines and purified using protein G affinity chromatography. IV.3 antibody (mouse monoclonal anti-human FcγRIIA) was produced in house. F(ab’)_2_ and Fab fragments of AYP1 and IV.3 were generated using Pierce^TM^ Fab and F(ab’)_2_ preparation kits (Thermo-Fisher Scientific) according to the manufacturer’s instructions. Purity of the fragments was assessed by SDS-PAGE and western blot with their functionality validated using flow cytometry, NFAT and LTA assays.

### Surface plasmon resonance binding studies

Surface plasmon resonance experiments were performed using a Biacore T200 instrument (GE Healthcare). For LUAS experiments, CLEC-2 (ECD 55-229) was immobilised directly onto the CM5 chip using amine-coupling to the carboxylmethylated dextran-coated surface. Reference surfaces were blocked using 1 M ethanolamine pH 8. Each concentration of analyte (LUAS, LUAS-2 and LUAS-2-Fc) was run as follows; 120 s injection, 300 s dissociation and a 120 s stabilisation period.

For AYP1 experiments, AYP1 Fab was immobilised directly onto the CM5 chip using amine-coupling to the carboxylmethylated dextran-coated surface. Each concentration of analyte (CLEC-2 ECD 53–229) was run as follows; 120 s injection, 300 s dissociation, 30 s regeneration with 10 mM Glycine pH 1.5 followed by a 300 s stabilisation period.

All sensograms shown are double reference subtracted and at least two replicates were injected per cycle as well as experimental replicates of *n* = 3. Experiments were performed at 25 °C with a flow rate of 30 μL/min in HBS-EP running buffer (0.01 M HEPES pH 7.4, 0.15 M NaCl, 3 mM EDTA, 0.005% v/v surfactant P20). Multi-cycle kinetic assays were used with at least five concentration points between 0.1× and 10× the KD. Kinetic analysis was performed using the Biacore T200 Evaluation software using a global fitting to a 1:1 binding model.

### Fluorescence correlation spectroscopy (FCS) and analysis

HEK293T cells were seeded in phenol red-free DMEM at a density of 3 × 10^4^ cells/coverslip onto cleaned 25 mm coverslips (Marienfeld, high precision, thickness No. 1.5H [0.170 mm ± 0.005 mm]) (details of cleaning procedure is in Supplementary Methods Section). The following day, cells were transiently transfected with PEI reagent in serum-free DMEM (phenol red-free) according to manufacturer’s instructions (PEI:DNA ratio = 3:1; 3 μg:1 μg) where 100 ng CLEC-2-eGFP DNA, 500 ng CD28-eGFP or 100 ng CD86-eGFP were used to achieve optimal receptor density. Cells were left to grow a further 24 h at 37°C/5% CO_2_. FCS measurements were made using a Zeiss LSM-880 inverted microscope equipped with gallium arsenide phosphide photon detectors (GaAsP) (Carl Zeiss, Jena, Germany) as reported^25^. Single-photon excitation with a continuous argon ion laser was performed using a 40x (NA 1.2) C-apochromat water immersion objective. Before each experiment, the microscope was aligned and calibrated using Atto-488 dye as reported^25^. FCS measurements were taken on the plasma membrane where monitoring the photon counts per molecule in real-time (interactive counts/molecule window in the Zeiss software) was performed to achieve optimum positioning in the centre of the observation volume and to find the focal plane corresponding to the maximal photon counts/molecule. Measurements were performed with 0.03% 488 nm laser power at 25°C for 5 s measurement time per point for 10 points per cell using Zen Black 2012 (Carl Zeiss, Jena, Germany).

LUAS nanobody (10 nM), LUAS-2 (10 nM) and LUAS-4 (10 nM) were diluted in phenol red-free DMEM and added to the cells and imaged immediately. For PRT samples, PRT-060318 (10 μM) was diluted in phenol red-free DMEM and added to cells for 45 min prior to ligand addition and imaging. FCS data were analysed with autocorrelation analysis to determine diffusion coefficients and photon counting histogram (PCH) analysis to determine molecular brightness and number of fluorescent molecules using Zen 2012 (black edition) software (Carl Zeiss, Jena, Germany) as reported^25^.

### Preparation of human-washed platelets for aggregometry

Blood was collected in trisodium citrate [1 part 3.8% (w/v) stock: 9 parts blood] from healthy and consenting volunteers by venipuncture in accordance with the Declaration of Helsinki (local ethical review no: ERN_11-0175). Washed platelets were prepared by centrifugation in the presence of prostacyclin (2.8 μM) followed by resuspension in Tyrodes-HEPES buffer (134 mM NaCl, 0.34 mM Na_2_HPO_4_, 2.9 mM KCl, 12 mM NaHCO_3_ 20 mM HEPES, 1 mM MgCl_2_, 5 mM glucose, pH 7.3) as reported^59^. Platelets were left for 30 min. For aggregation measurements, platelets were used at 2 × 10^8^/ml. Aggregation was monitored in a Chronolog model 700 aggregometer (ChronoLog, Havertown, PA, USA) at 37 °C with constant stirring at 1200 rpm. Platelets were pre-treated with anti-FcγRIIA IV.3 Fab (30 µg/ml), PRT-060318 (1 µM), PP2 (20 µM), AYP1 F(ab’)_2_ (66 nM), LUAS-2 (1-600 nM) or vehicle [0.1% (v/v) DMSO] for 5-10 min. Platelets were stimulated with AYP1 mAb (66 nM), AYP1 F(ab’)_2_ (66 nM), AYP1 F(ab) (66 nM), rPDPN-Fc (136 nM), WT-HEK293T (5×10^5^ cells), PDPN-KO HEK293T (5 × 10^5^ cells), LUAS (10 or 450 nM), LUAS-2 (10 or 450 nM), LUAS-2-Fc (1.25 nM), LUAS-4 (10 nM) or rhodocytin (100 nM) and aggregation was monitored for 10 min. For aggregations with LUAS-2, platelets were pre-incubated with hirudin (75 μg/ml) to block potential thrombin trace contamination introduced during nanobody his-tag cleavage.

### Humanised CLEC-2 mouse model and aggregometry

The generation of the humanised CLEC-2 mouse (hCLEC-2^KI^) has been reported^29^. WT C57BL/6 mice were purchased from Charles River Laboratories. Humanised CLEC-2 (hCLEC-2^KI^) mice were generated by Biocytogen on a C57Bl/6 N background using CRISPR Cas9 to replace the mouse Clec1b gene with the human variant. Mice were aged between 12 and 14 weeks; males and females. There was no sex or age-specific analysis performed in the animal studies. Mouse hCLEC-2^KI^ washed platelets were prepared as reported^29^. Platelet aggregation was measured using a 4- channel aggregometer (APACT) under stirring conditions for 10 min after the addition of LUAS-2 (1–100 nM) or LUAS-4 (1–10 nM). For aggregations with inhibitors, the platelets were preincubated for 5 min at 37 °C with either saline, AYP1 Fab (208 nM) or the Syk inhibitor Bl1002494 (500 nM). Animal studies were either approved by the district government of Lower Franconia or were performed in accordance with the Animal (Scientific Procedures) Act 1986 with approval of the UK Home Office under PPL PP9677279 and P06779746 granted to the University of Birmingham.

### NFAT luciferase reporter assay

CLEC-2-eGFP (details in Supplementary Materials) DNA was used at 100 ng or 2 µg in combination with 15 μg NFAT-luciferase reporter DNA. DT40 cells (2 × 10^7^/transfection) were transfected with CLEC-2 and NFAT luciferase reporter in serum-free RPMI by electroporation as described in Supplementary Methods Section. The following day, cells were incubated with rhodocytin (30 nM), AYP1 mAb (6.6 nM), AYP1 F(ab’)_2_ (9.1 nM), AYP1 Fab (20.8 nM), LUAS nanobody (10 nM), LUAS-2 (10 nM), LUAS-2-Fc (10 nM) or positive controls PMA (50 ng/ml) and ionomycin (1 μM) or RPMI for 6 h at 37°C and frozen at −80 °C. For antagonism experiments, LUAS-2 (3 μM) was incubated with the cells for 30 min prior to rhodocytin (30 nM) addition. The next day, cells were harvested using luciferase harvest buffer (1 M KH_2_PO_4,_ 12.5% Triton X-100 and 1 M dithiothreitol) and added to luciferase assay buffer (1 M KH_2_PO_4,_ 0.1 M MgCl_2_, 0.1 M ATP in ddH_2_O) in a white, opaque 96-well plate. Luciferase activity was measured with a Centro LB 960 microplate luminometer (Berthold Technologies Wildbad, Germany). The machine was primed with luciferin substrate (1 mM) made in ddH_2_O followed by sequential injection of luciferin into the wells (50 μl, counting time 10 s per well). Transfection success of CLEC-2 was assessed by flow cytometry with AYP1 mAb as described in Supplementary Methods Section.

### Single-molecule stepwise photobleaching and analysis

HEK293T cells were seeded on clean coverslips as described above. Cells were transiently transfected with PEI reagent in SFM as described above where 0.5 ng CLEC-2-eGFP, 10 ng CD28-eGFP and 2 ng CD86-eGFP DNA were used to achieve low receptor density. Empty pCI-neo vector was used to ensure total cDNA concentrations were consistent across all transfections. Single-molecule photobleaching imaging was performed using an Nikon Eclipse Ti inverted microscope in TIRF mode with a Nikon 100×1.49 NA TIRFM oil objective, Perfect Focus System, Agilent MLC400 laser bed with 405 nm (50 mW), 488 nm (80 mW), 561 nm (80 mW) and 640 nm (125 mW) solid-state lasers and Andor iXon Ultra EM-CCD camera. During the acquisition, at 28°C the sample was continuously illuminated at 488 nm using a 488 TIRF filter for 5000 frames with 8% laser power (40 × 40 μm, 30 ms exposure time, 300 gain). To ensure homogenous illumination, a central 256 × 256 pixel region of interest of the chip was used. Microscope control and image acquisition were performed by NIS Elements 5 (Nikon Instruments).

Trace extraction and stepwise photobleaching analysis was performed using the python package quickpbsa^36^ as reported^25^ (details in Supplementary Methods Section). Receptor density (localisations/μm^2^) was calculated by dividing the number of localisations by the cell area (Supplementary Fig. 4). Further information about modelling is in Supplementary Methods Section.

### Generation of SMALPs from human platelets and SMA-PAGE

Washed human platelets were prepared following the Watson protocol as described above. Platelets were resuspended in modified Tyrode’s buffer (134 mM NaCl, 0.34 mM Na_2_HPO_4_, 2.9 mM KCl, 12 mM NaHCO_3_, 20 mM HEPES, 5 mM glucose, 1 mM MgCl_2_, pH 7.3) at 4 × 10^8^/ml, centrifuged down at 10,000 × *g* and resuspended in SMALP buffer (50 mM Tris pH = 8, 150 mM NaCl, 10% glycerol) to the same concentration. Platelets in solution were homogenised with a hand homogeniser until no cell clumps were visible. The SMA polymer, SMALP 30010 P (S:A = 2.3:1, Orbiscope, the Netherlands), was added into the homogenised platelet solution with a final concentration of 2.5% (wt/vol), and the platelet-SMA mixture was incubated at room temperature for 2 h. After incubation, the mixture was ultracentrifuged in a SW60 Ti rotator at 165,051 *g* for 1 h at 4 °C. The supernatant was collected, NaCl supplemented to 500 mM to avoid non-specific aggregation of SMALPs, and subjected to SMA-PAGE.

The SMA-PAGE protocol was established as described^39^ using Tris-Glycine buffer system. Briefly, SMALPs in solution were mixed with native sample buffer (20 mM Tris pH 8, 50% (v/v) glycerol, 0.08 mg/ml bromophenol blue), and loaded directly into Novex WedgeWell Tris-Glycine gels (4–12% or 4–20%). Electrophoresis was performed at room temp, with SMA-PAGE running buffer (25 mM Tris pH 8.8, 192 mM glycine).

For Western blot analysis, the gel was then soaked in SDS-PAGE running buffer (25 mM Tris, 192 mM glycine, 0.1 % SDS) for 10 min with gentle agitation, to facilitate proteins transferring to the PVDF membrane by coating the negatively charged SDS on their surface. After that, the gel was briefly soaked in transfer buffer, and proteins transferred to PVDF membrane using an XCell II blot module, at 4 °C, 200 mA for 2 h. The membrane was blocked with 25 mM L-arginine and 5% BSA in TBST (with 0.1 % azide) for 1 h at room temp, then probed with mouse anti-CLEC-2 antibody AYP2 (made in house, 1:500) or rabbit anti-αIIb antibody (sc-15328, Santa Cruz, 1:500) in 5% BSA in TBST overnight at 4 °C. The probed membrane was washed 3 × 10 min in TBST, then probed with HRP-conjugated secondary antibody (anti-mouse or anti-rabbit, 1:5000 in TBST) at room temp for 1 h, followed by a further 3 × 10 min wash in TBST. The signal was detected by enhanced chemiluminescence reagents (Pierce ECL substrate, ThermoFisher), developing and fixing reagents.

### Statistical and reproducibility

Results are shown as mean ± SD unless otherwise stated and the number of independent experiments is described in Figure legends. Data were analysed using PRISM v9.2.0 (GraphPad, San Diego, CA). For FCS, data sets were first tested for normality using the Shapiro-Wilks test. FCS data were tested by Kruskal-Wallis with Dunn’s post-hoc test. For stepwise photobleaching experiments, statistical analysis was by Epps-Singleton 2 sample test^60^ implemented in scipy^61^. Statistical analysis of the aggregations and flow cytometry was by a one-way ANOVA with a Bonferroni *post-hoc* test. For NFAT assays, Student’s two-tailed unpaired *t* tests were used. Significance was set at *P* ≤ 0.05.

### Reporting summary

Further information on research design is available in the Nature Portfolio Reporting Summary linked to this article.

## Supplementary information

Supplementary Information FINAL

Description of Additional Supplementary Data

Supplementary Data 1

Reporting Summary

## Data Availability

All data are available in the main text or the Supplementary Materials including Supplementary Fig. 6 and Supplementary Data 1. All other data are available from the corresponding author upon reasonable request.

## Code availability

The quickPBSA package, example data, and documentation are available at https://github.com/JohnDieSchere/quickpbsa.
