# Supplementary material for: Divalent nanobodies to platelet CLEC-2 can serve as agonists or antagonists
Source: Commun Biol. 2023 Apr 7;6:376. doi: 10.1038/s42003-023-04766-6 (PMC10082178; doi:10.1038/s42003-023-04766-6)
Supplement: Supplementary file 1 — Supplementary Information FINAL [file 42003_2023_4766_MOESM1_ESM.pdf]

Supplementary Information

Divalent nanobodies to platelet CLEC-2 can serve as agonists or antagonists

Joanne C. Clark[1,2]†*, Eleyna M. Martin[1]†, Luis A. Morán[1,3]†, Ying Di[1], Xueqing Wang[1,3,4], Malou Zuidscherwoude[1,2], Helena C. Brown[1,5], Deirdre M. Kavanagh[6], Johan Hummert[1,2], Johannes A. Eble[7], Bernhard Nieswandt[5], David Stegner[5], Alice Y. Pollitt[8], Dirk-Peter Herten[1,2], Michael G. Tomlinson[2,4], Angel García[3], Steve P. Watson[1,2]*

[1]Institute of Cardiovascular Sciences, Level 1 IBR, College of Medical and Dental Sciences, University of Birmingham, Edgbaston, Birmingham, B15 2TT, UK.

[2]Centre of Membrane Proteins and Receptors (COMPARE), The Universities of Birmingham and Nottingham, The Midlands, UK.

[3]Centre for Research in Molecular Medicine and Chronic Diseases (CIMUS), Universidade de Santiago de Compostela, and Instituto de Investigación Sanitaria de Santiago (IDIS), Santiago de Compostela, Spain.

[4]School of Biosciences, University of Birmingham, Edgbaston, Birmingham, B15 2TT, UK.

[5]Institute of Experimental Biomedicine I, University Hospital and Rudolf Virchow Center for Integrative and Translational Bioimaging, University of Würzburg, Würzburg, Germany.

[6]Department of Biochemistry, University of Oxford, South Parks Road, Oxford, OX1 QU3, UK.

[7]Institute for Physiological Chemistry & Pathobiochemistry, University of Münster, Waldeyerstraße 15, 48149 Münster, Germany.

[8]Institute for Cardiovascular and Metabolic Research, School of Biological Sciences, University of Reading, Reading, RG6 6AS, UK.

†These authors contributed equally to this study.

***Corresponding authors:** Dr. Joanne C Clark, Email: j.clark.5@bham.ac.uk**;** Prof. Steve P Watson, Email: S.P.Watson@bham.ac.uk

Institute of Cardiovascular Sciences, Level 1 IBR, College of Medical and Dental Sciences, University of Birmingham, Edgbaston, Birmingham B15 2TT, UK. Tel: +44 (0) 121 414 6514

Construct generation

All constructs were sequenced for correctness.

CLEC-2-eGFP (A206K): To make a N-terminally eGFP tagged CLEC-2 construct, human CLEC-2 with the linker (DRNLPPLAPL) DNA was synthesised (GeneArt, Invitrogen) and subcloned in the pEGFP(A206K)-C1 expression vector for expression of CLEC-2-eGFP where the eGFP has a A206K mutation to prevent dimerisation of the fluorescence protein.

CD28 and CD86-eGFP (A206K): DNA coding for CD28 and CD86 was kindly provided by Davide Calebiro (University of Birmingham, UK) and was digested with *XhoI* and *BamHI* restriction enzymes and inserted into similar cut pEGFP-N1 vector.

NFAT-luciferase reporter: The construct has been reported.[1]

Antibodies

Syk p525/526 pAb was from Cell Signaling Technology (Massachusetts, United States). LAT p200 mAb was obtained from Abcam (Cambridge, UK). Mouse anti-human anti-phosphotyrosine (clone 4G10) mAb was from Millipore UK Ltd (Watford, UK).

Generation of CLEC-2 nanobodies

Nanobodies targeted against the extracellular domain of platelet receptor CLEC-2 were generated in collaboration with VIB Nanobody Core (Belgium). In short, immunisation of a llama occurred by subcutaneous injection of 100 µg recombinant human CLEC-2 (residues 55-229 with an N-terminal His_6-tag) on days 0, 7, 14, 21, 28 and 35. Anti-coagulated blood was collected for lymphocyte preparation on day 40. From the lymphocytes a VHH library was constructed consisting of more than 108 independent transformants, with 81% harbouring the right size insert in the phagemid vector (pMECS-GG). Three rounds of panning on solid-phase coated CLEC-2 antigen (100 µg/mL) were performed with enrichment for antigen-specific phages assessed after each round. Subsequently, 285 colonies were analysed by ELISA for the presence of CLEC-2 antigen specific nanobodies in crude periplasmic extracts. The screening revealed 107 positive colonies of which 48 represented unique nanobody sequences belonging to 12 different CDR3 groups. The library of clones was provided by VIB as E. coli TG1 harbouring phagemid pMECS-GG containing nanobody genes.

Production of CLEC-2 nanobodies in E. coli

CLEC-2 Nbs received in the pMECS-GG vector contain an N-terminal PelB signal sequence that targets the nanobody to the perisplasmic space of *E. coli* allowing their extraction from the periplasm. All 48 Nbs were expressed from small scale TG1 *E. coli* cultures for initial screening. Selected Nbs were then expressed from the WK6 *E. coli* strain (provided by VIB) as follows. WK6 colonies transformed with CLEC-2 Nb construct were grown overnight at 37°C in LB broth containing 100 µg/ml ampicillin. Overnight cultures were used to inoculate 1L TB medium (2.3g/L KH_2PO_4, 16.4 g/L $K_2HPO_4.3H_2O$, 12 g/L Tryptone, 24 g/L Yeast Extract, 4 mL/L Glycerol) 1:300 dilution supplemented with 100 µg/ml ampicillin. Cultures were incubated at 37°C, 180 rpm until an OD590 of 0.6-0.9 was reached. Nanobody expression was then induced with addition of β-D-thiogalactoside (IPTG) to a final concentration of 1 mM. Cultures were left to express for 16 h at 28°C, 180 rpm. Nanobody containing *E. coli* cells were harvested by centrifugation for 15 min at 3000 rpm, the supernatant was discarded and the cell pellet resuspended in TES buffer (0.2 M tris pH 8, 0.5 mM EDTA, 0.5 M sucrose), 12 mL per 1 L culture, incubated with gentle agitation at 4°C for one h. The cell suspension was diluted 2x in TES/4 (4x dilution of TES buffer) and left to incubate at 4°C for one further h. Insoluble protein and cell debris was removed from the suspension by centrifugation at 8000 rpm for 30 min, 4°C leaving the supernatant containing nanobody and any other proteins extracted from the periplasmic space. Nanobodies were purified from the periplasmic extract using nickel-coated beads affinity columns followed by size exclusion chromatography on a HiLoad 26/600 Superdex 75 pg column (Cytiva). The concentration of purified nanobody was determined via Nanodrop measuring absorbance at 280 nm according to the manufacturer's protocol. For his-tag cleavage of LUAS-2, thrombin (1 unit/ml) was added to LUAS-2-bound his-tag beads and incubated overnight at room temp. The following day, AEBSF (20 mg/ml stock) was added and incubated for 10 min at 4°C to inactivate residual thrombin.

Production of LUAS-2-Fc in mammalian cells

HEK293T cells were cultured at 37°C and 5% CO2 in complete DMEM (Dulbecco's Modified Eagle's Medium supplemented with 10% fetal bovine serum, 1% penicillin, 1% streptomycin and 1% glutamine). LUAS-2-Fc DNA was transiently transfected into

mammalian HEK293T cells at 60% confluency using polyethylenimine (PEI Max MW 40,000, Polysciences) according to the manufacturer's instructions and expressed as secreted protein, due to the presence of an N-terminal secretion signal, into the culture media. LUAS-2-Fc secreted into the medium was collected after four days and subsequently purified using protein A affinity chromatography followed by size exclusion chromatography on a HiLoad 26/600 Superdex 75 pg column (Cytiva). The concentration of purified nanobody was determined via Nanodrop measuring absorbance at 280 nm according to the manufacturer's protocol.

CLEC-2 and podoplanin protein expression and purification

The cDNA sequences for the extracellular domain of human CLEC-2 (residues 55-229) and podoplanin were cloned into pHLSEC (Adegene) and pFUSE-Fc (InvivoGen) expression vectors respectively. Both constructs were expressed in house and sequenced for correctness. Recombinant human his6-CLEC-2 and recombinant human podoplanin-rFc were expressed in HEK293T cells by transient transfection using PEI (PEI Max MW 40,000) in serum free DMEM. After 4 days, proteins secreted into the medium were collected. Recombinant human his6-CLEC-2 and podoplanin-rFc were purified in a gravity purification column using Nickel-NTA resin (ThermoFisher) and protein A-coated beads (ThermoFisher) respectively. SDS-PAGE was used to confirm protein purity.

CRISPR-Cas9–mediated podoplanin knockout in HEK293T cells

Plasmid based CRISPR-Cas9 gene editing was used to knockout podoplanin expression in HEK293T cells.[2] Briefly, guide sequences targeting exon 1 of the human podoplanin gene were identified using the Sanger CRISPR Finder and selected on the least potential off-target binding.[3] Guide sequences, as complementary oligos (5'-CACCGACAACTCAACGGGAACGATG-3' and 5'-AAACCATCGTTCCCGTTGAGTTGTC-3') were annealed and phosphorylated using T4 Polynucleotide Kinase, followed by ligation into *BbsI* digested pSpCas9(BB)-2A-Puro. The plasmid was transfected into HEK293T cells and clonal cells selected for using 2 µg/ml of puromycin. Loss of podoplanin expression was confirmed using flow cytometry and Western blotting.

Cell culture and transfection

Cell lines were purchased from ATCC. Wild type and podoplanin-null HEK293T cells were cultured at 37°C and 5% CO_2 in DMEM supplemented with 10% fetal bovine serum, 1% penicillin, 1% streptomycin and 1% glutamine. DT40 chicken B-cells were cultured at 37°C and 5% CO_2 in RPMI supplemented with 10% fetal bovine serum, 1% penicillin, 1% streptomycin, 1% glutamine, 1% chicken serum and 50 µM 2β-mercaptoethanol. For transfection of HEK293T cells, polyethylenimine (PEI) was used at a DNA to PEI ratio of 1 µg: 3 µl in non-supplemented DMEM. For transfection of DT40 cells, cells were electroporated in the presence of plasmid DNA in non-supplemented RMPI at 0.350kV and 0.500 F (GenePulser II, Bio-Rad).

Platelet lysis and protein phosphorylation

For Western blotting, whole platelet lysates ($5x10^8$/ml) were prepared as reported.[4] Platelets were pre-treated with PRT-060318 (1 µM), PP2 (20 µM), AYP1 $F(ab)_2$ (66 nM) or vehicle [0.1% (v/v) DMSO] as control for 5 min. Platelets were stimulated with rhodocytin (100 nM) or LUAS-4 (10 nM). Platelet lysates were electrophoresed on 4-12% BisTris Plus acrylamide gels (Invitrogen, Paisley, UK) and immunoblotted on PVDF membranes as reported.[4] Membranes were incubated with primary antibodies, anti-phosphotyrosine (clone 4G10; 1:1,000), anti Syk Tyr 525/526 (1:500) and anti-LAT Tyr 200 (1:500). Bands were visualised by film.

Humanised CLEC-2 mouse platelet aggregometry

Mouse hCLEC-2^{KI} washed platelets were prepared as described in the main text. For aggregation measurements, platelets were used at $2x10^8$/ml. Aggregation was monitored in a Chronolog model 700 aggregometer (ChronoLog, Havertown, PA, USA) at 37ºC with constant stirring at 1200 rpm. Platelets were preincubated with AYP1 Fab (66 nM) for 5 min at 37ºC prior to stimulation with LUAS-2 (100 nM) or LUAS-2-Fc (10 nM). Aggregation was monitored for 5 min.

Fluorescence correlation spectroscopy (FCS)

On the day of the experiment, the FCS confocal volume was determined by measurement of the axial and lateral radii. The axial and lateral radii were 1.79+0.93 µm and 0.22+0.03 µm respectively and the confocal volume was 0.19+0.04 $µm^3$ (0.19 fl) (Supplementary Figure 1A). Coverslips were acid cleaned with 1M HCl followed by 100% ethanol and air dried.

Single molecule photobleaching analysis and modelling

Fluorescent spots were localised using the ImageJ[5] plugin ThunderSTORM[6] on the average of the first 5 frames in the sequence, extracting the spots centre position and Gaussian width (σ). Spots with a σ below 50 nm and above 250 nm, or within a distance of 3.5 pixels (576 nm) of another spot, were excluded. Image sequences with aberrant imaging conditions were excluded based on the overall fluorescent decay. Photobleaching traces were extracted from a ROI with a 2 pixel radius (329 nm). A ring-shaped ROI with an inner radius of 3.5 pixels (576 nm) and an outer radius of 5 pixels (832 nm) was used for background subtraction. Photobleaching analysis in quickpbsa was run with a step detection threshold of 20 counts and otherwise default parameters. However, to increase the robustness to photophysics the Bayesian refinement step in the quickpbsa analysis was not used, since this step relies on reproducible brightness of a single fluorophore and could be affected by blinking. This approach is valid for small fluorophore numbers (<10).[7]

The eGFP number distributions obtained for CD86 and CD28 were modelled with binomial distributions using least-squares fitting from the optimised module of the python package scipy[8] (Supplementary Figure 3). For CD86, the model function is:

(1)

$$P(k, r_{rc})_{mononer} = \binom{1}{k}(1 - r_{rc}) + \binom{2}{k} r_{rc}$$

With the fraction of double spots r_{rc} as a free parameter. For the monomers the labelling efficiency is set to 100%, since unlabelled monomers cannot be detected.

For CD28, we assume that CD28 is fully dimerised and that some CD28 dimers are randomly co-localised within one diffraction-limited volume, which would explain the observation of bright spots containing 3 or 4 eGFP copies. The model function is then given as the sum of two binomial probability distributions:

(2)

$$P(k, r_{rc}, p_{le})_{dimer} = \binom{2}{k} p_{le}^{k}(1 - p_{le})^{(2-k)(1-r_{rc})} + \binom{4}{k} p_{le}^{k}(1 - p_{le})^{(4-k)} r_{rc}$$

with the fraction of double spots r_{rc} and the labelling efficiency p_{le} as free parameters. The model yields a labelling efficiency of 58%. The labelling efficiency could be

underestimated due to blinking or unresolved steps in the analysis. The fraction of double spots in the model is 32%, which is consistent with the fraction of double spots observed in the measurement with the purely monomeric CD86 (24%).

The eGFP number distribution obtained for CLEC-2 was modelled with a binomial distribution using least squares fitting with python scipy.[8] For the mixture of monomers and dimers, the model function is:

(3)

$$P(k, r_d)_{mix} = (1 - r_d)P(k)_{monomer} + r_d P(k)_{dimer}$$

with the fraction of double spots r_{rc} and the labelling efficiency p_{le} as fixed parameters which were extracted from the modelling of CD28 data. The model yields a fraction of dimers of 64%.

Flow cytometry to measure CLEC-2 expression in cell lines

To measure expression of the CLEC-2-eGFP construct used in NFAT assays on the surface of DT40 cells or HEK293T for FCS experiments, a sample of each transfection was stained with AYP1 anti-CLEC-2 antibody (66 nM) followed by anti-mouse Alexa Fluor-647 secondary antibody staining (1:400). The samples were acquired and analysed in an Accuri C6 flow cytometer (BD Biosciences, USA). Cell populations were gated on cell size using forward scatter (FSC) vs side scatter (SSC) to distinguish them from electronic noise. The light scatter and fluorescent channel (FL4) were set to logarithmic gain and 10,000 events per sample were analysed. Data expressed as MFI (a.u) and histograms were made using cSampler Software (BD Biosciences, USA).

Flow cytometry to measure CLEC-2 expression in human platelets and humanised hCLEC-2^{KI} mouse platelets

To measure expression of human CLEC-2 on the surface of human and hCLEC-2^{KI} mouse platelets, the platelets were stained with AYP1 anti-hCLEC-2 antibody (66 nM) followed by anti-mouse Alexa Fluor-647 secondary antibody staining (1:400) at room temp. Control samples with no staining and secondary antibody staining only were also analysed. The samples were acquired (FL4) and analysed as described above. Histograms were made in cSampler Software (BD Biosciences, USA).

CLEC-2 nanobody binding with flow cytometry

To investigate CLEC-2 nanobody binding with flow cytometry washed platelet samples were incubated with the nanobodies (7 nM) for 15 min at room temp followed by Alexa Fluor-647 anti-6-His tag antibody secondary labelling (5 µg/ml). Control samples with no staining and secondary antibody staining only were also analysed. The samples were acquired (FL4) and analysed as described above. Histograms were made in cSampler Software (BD Biosciences, USA).

Supplementary Figures

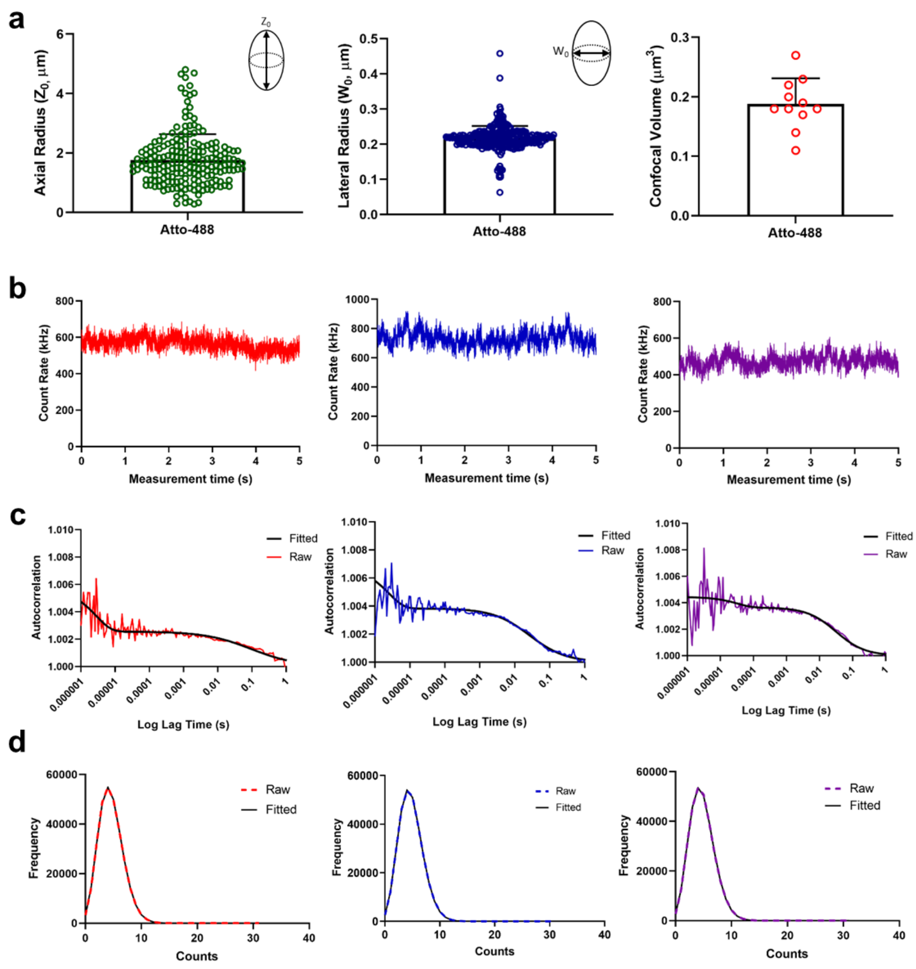

Supplementary Figure 1. Calibration of confocal volume with Atto-488 dye. (a) On the day of each FCS experiment, the confocal microscope was calibrated using Atto-488 dye (10 nM) in water at 25°C with a known diffusion coefficient of 400 $\mu m^2 s^{-1}$ to determine axial and lateral radii and confocal volumes. Raw autocorrelation data for Atto-488-dye was fitted with 1-component 3D, free diffusion autocorrelation model. The axial (Z_o) and lateral (W_o) radii were determined to be ~1.79±0.93 µm and ~0.22±0.03 µm respectively and the confocal volume was ~0.19±0.04 μm^3. At least 190 FCS calibration measurements were taken. Data are presented as mean±SD. (n=19 biologically independent experiments). (b) Representative raw fluorescence intensity fluctuation traces for eGFP-tagged CD86 (red), CD28 (blue) and CLEC-2 (purple) shown in Figure 7c. (c) Representative raw autocorrelation data for CD86 (red), CD28 (blue) and CLEC-2 (purple) with the autocorrelation 1-component fits (black curves). (d) Representative raw PCH distributions for CD86 (dotted red line), CD28 (dotted blue line) and CLEC-2 (dotted purple line) with 1-component PCH model fitting (black lines). FCS measurements were taken in 53-70 cells (n=3-6 19 biologically independent experiments).

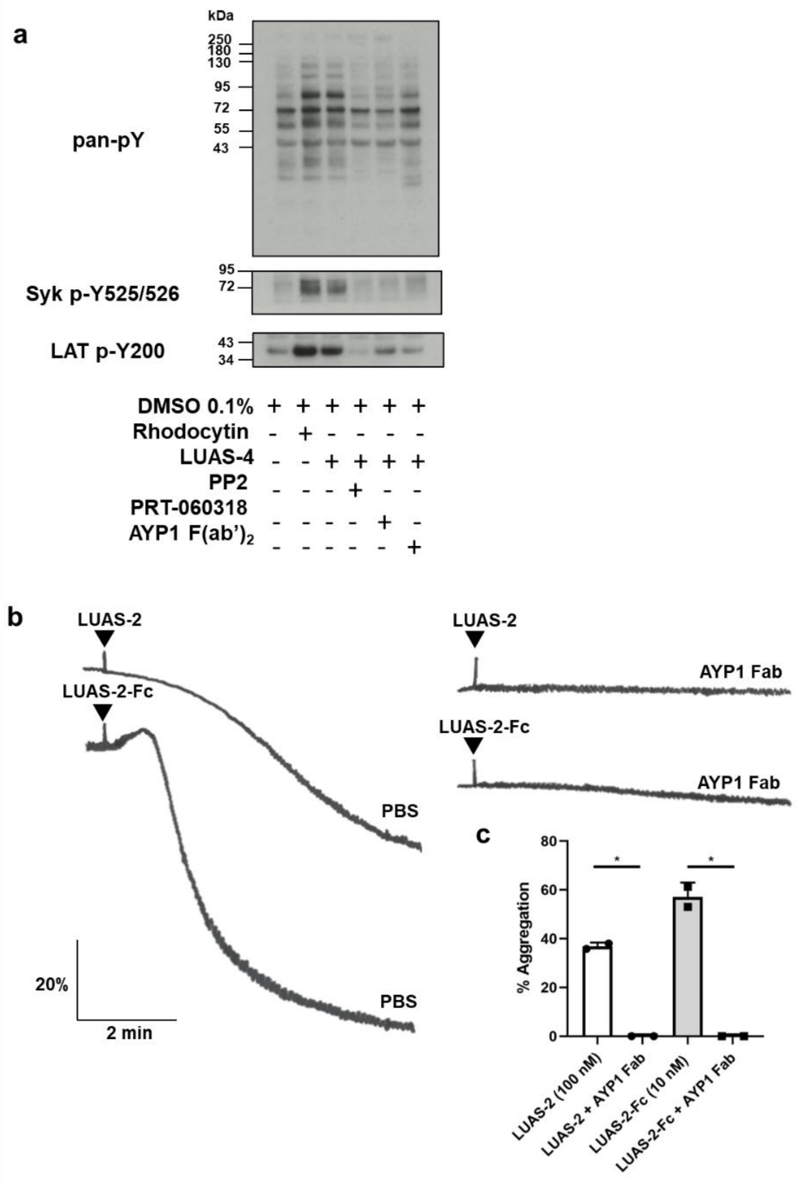

Supplementary Figure 2. Divalent LUAS-2 and tetravalent LUAS-4 or LUAS-2-Fc induce platelet activation via CLEC-2. (a) The effect of PRT-060318 (1 µM), PP2 (20 µM) and AYP1 F(ab')$_2$ (66 nM) on LUAS-4 (10 nM) induced phosphorylation in washed platelets was investigated by western blotting with the phosphotyrosine antibody 4G10 and phosphospecific antibodies to Syk and LAT. Rhodocytin (100 nM) was used as a control (n=3 biologically independent experiments). (b-c) Humanised CLEC-2 mouse (*hCLEC1b^{KI}*) platelet aggregation was monitored by light transmission aggregometry at 37°C with constant stirring at 1200 rpm for 5 min. (b) Representative traces of washed hCLEC-2^{KI} platelet aggregation (2x10^8 platelets/ml) induced by LUAS-2 (100 nM), LUAS-2-Fc (10 nM), by LUAS-2 (100 nM) + AYP1 Fab (66 nM) and LUAS-2-Fc (10 nM) + AYP1 Fab (66 nM). Platelets were preincubated with AYP1 Fab for 5 min at 37°C prior to stimulation. (c) Percentage of platelet maximal aggregation induced by LUAS-2 (100 nM) and LUAS-2-Fc (10 nM) in the absence and presence of AYP1 Fab (66 nM) (n=2 biologically independent experiments). Significance was measured with a one-way ANOVA with Bonferroni *post-hoc* for where $P \leq 0.05$. Data are presented as mean±SD.

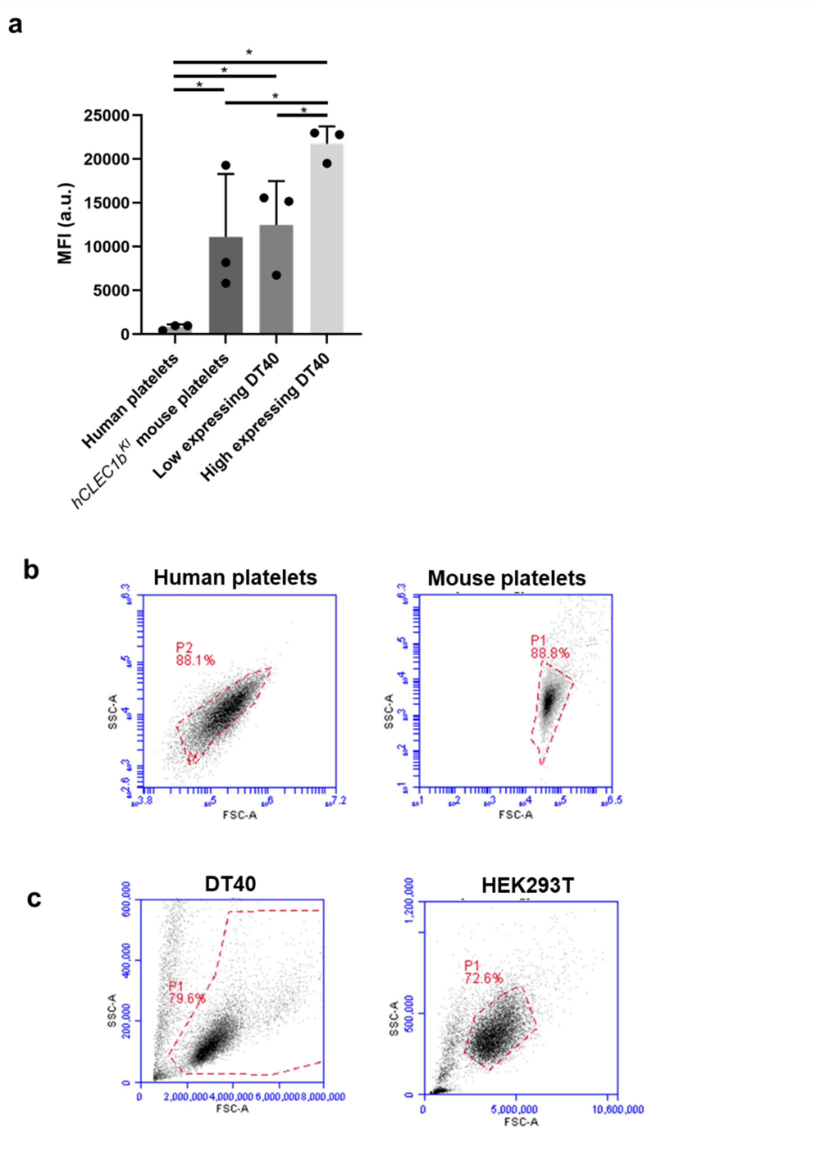

Supplementary Figure 3. Comparison of expression levels of CLEC-2 in human platelets, humanised CLEC-2 mouse platelets (*hCLEC1b*^{KI}), low and high expressing transfected DT40 cells. (a) Expression of human CLEC-2 in washed human platelets (2x10^8 platelets/ml), washed humanised CLEC-2 mouse platelets (*hCLEC1b*^{KI}) (2x10^8 platelets/ml), Low expressing (100 ng DNA) and high expressing (2 µg DNA) transfected DT40 chicken B cells was measured by flow cytometry using the anti-hCLEC-2 AYP1 antibody (66 nM) with anti-mouse Alexa Fluor-647 secondary staining. Flow cytometry data presented MFI (n=3 biologically independent experiments). For transfected DT40 cells the CLEC-2-eGFP construct was used. Significance was measured with a one-way ANOVA with Bonferroni *post-hoc* for where $P \leq 0.05$. Data are presented as mean±SD. (b-c) Gating strategies for flow cytometry experiments. (b) Representative scatter dot plot showing the gating strategy for human and humanised mouse washed platelets for experiments in Figure 1a-b & Supplementary Figure 3a. (c) Representative scatter dot plot showing the gating strategy for transfected DT40 or HEK293T cells for experiments in Figure 6a-b and Figure 9a.

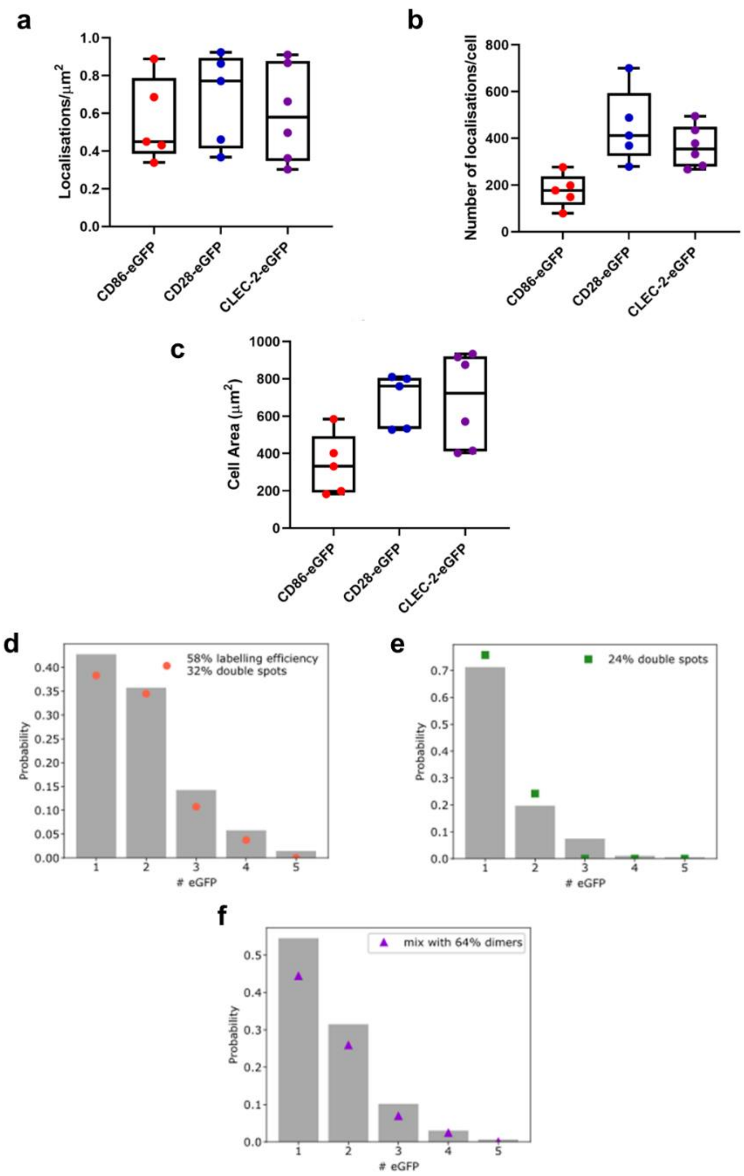

Supplementary Figure 4. Stepwise photobleaching modelling. (a) The density of CD86-eGFP, CD28-eGFP and CLEC-2-eGFP. The density of CD86, CD28 and CLEC-2 receptors (localisation/μm^2) was calculated by dividing the number of localisation by the cell area. (b) Number of localisations/cell for CD86, CD28 and CLEC-2. Fluorescent spots were localised using the ImageJ plugin ThunderSTORM on the average of the first five frames in the sequence, extracting the spots' centre position and Gaussian width (σ). (c) The HEK293T cell area (μm^2)/ROI measured using ImageJ. (d) Modelling of photobleaching step frequency histogram of CD28-eGFP (assuming it is always a dimer) determined the labelling efficiency of eGFP and the fraction of unresolved double spots in the photobleaching experiments. (e) Modelling of photobleaching step frequency histogram of CD86-eGFP (assuming it is always a monomer) determined the fraction of unresolved double spots in the photobleaching experiments. (f) Modelling the CLEC-2-eGFP distribution with a mixture of monomers and dimers model yielded an estimation of 64% dimers. The labelling efficiency and fraction of unresolved double spots were fixed to CD28-eGFP measurements.

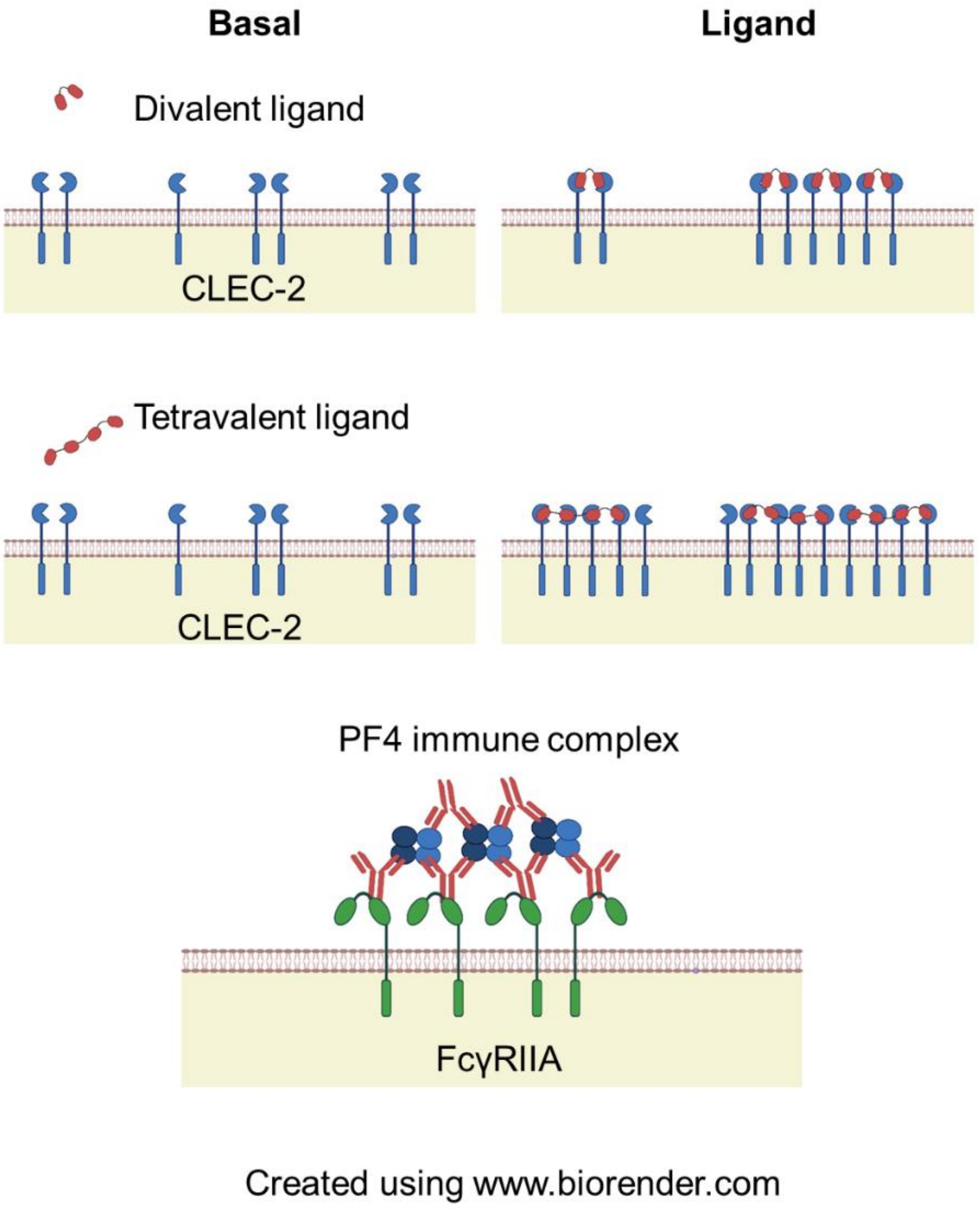

Supplementary Figure 5. Tetravalent ligands drive the formation of higher order clusters of CLEC-2 and FcgRIIA. A schematic diagram with top panels illustrating that the addition of a divalent or tetravalent ligand drives the formation of higher order clusters of CLEC-2 with the tetravalent ligand able to cause formation of much larger complexes. The bottom panel illustrates the clinical relevance of this mechanism in conditions such as vaccine-induced immune thrombocytopenia with thrombosis (VITT). The generation of high affinity antibodies to tetravalent platelet factor 4 (PF4) leads to tetrameric clusters of the Fc region of IgG which forms large clusters of FcgRIIA on the platelet surface.

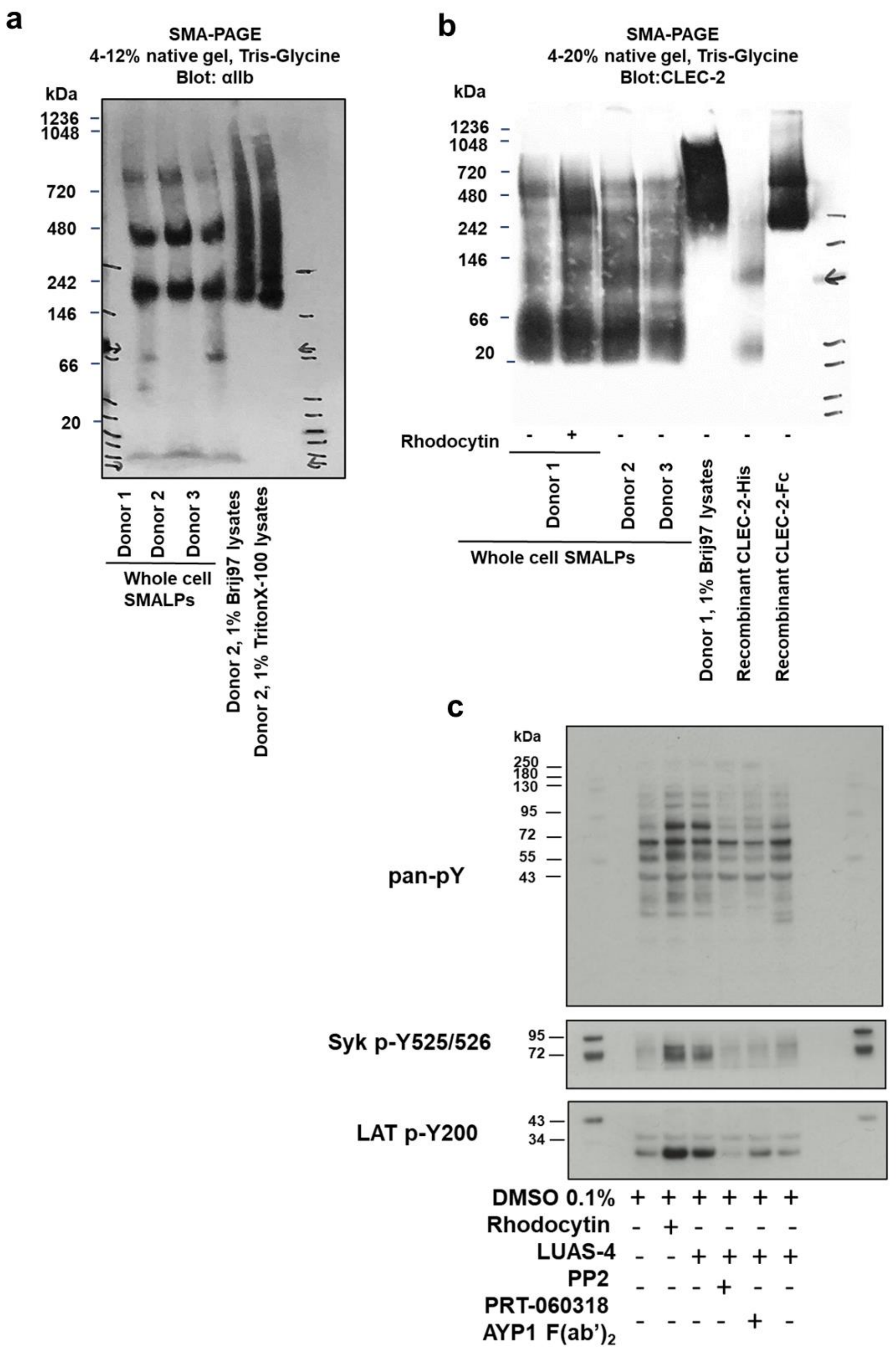

Supplementary Figure 6. Uncropped and unedited blot/gel images for (a-b) Figure 10 & (c) Supplementary Figure 2.

Supplementary References

1. Tomlinson MG, Calaminus SD, Berlanga O, Auger JM, Bori-Sanz T, Meyaard L and Watson SP. Collagen promotes sustained glycoprotein VI signaling in platelets and cell lines. J Thromb Haemost. 2007;5(11):2274-2283. 10.1111/j.1538-7836.2007.02746.x

2. Ran FA, Hsu PD, Wright J, Agarwala V, Scott DA, Zhang F. Genome engineering using the CRISPR-Cas9 system. Nat Proto. 2013;8(11):2281-308. 10.1038/nprot.2013.143

3. Hodgkins A, Farne A, Perera S, Grego T, Parry-Smith DJ, Skarnes WC, et al. WGE: a CRISPR database for genome engineering. Bioinformatics. 2015;31(18):3078-80. 10.1093/bioinformatics/btv308

4. Clark JC, Kavanagh DM, Watson S, Pike JA, Andrews RK, Gardiner EE, et al. Adenosine and Forskolin Inhibit Platelet Aggregation by Collagen but not the Proximal Signalling Events. Thromb Haemost. 2019;119(7):1124-37. 10.1055/s-0039-1688788

5. Schindelin J, Rueden CT, Hiner MC and Eliceiri KW. The ImageJ ecosystem: An open platform for biomedical image analysis. Mol Reprod Devt. 2015; 82(7-8):518-529. 10.1002/mrd.22489

6. Ovesný, M., Křížek, P., Borkovec, J., Svindrych, Z., & Hagen, G. M. ThunderSTORM: a comprehensive ImageJ plug-in for PALM and STORM data analysis and super-resolution imaging. Bioinformatics. 2014;30(16):2389-2390. 10.1093/bioinformatics/btu202

7. Hummert J, Yserentant K, Fink T, Euchner J, Ho YX, Tashev SA, et al. Photobleaching step analysis for robust determination of protein complex stoichiometries. Mol Biol Cell. 2021;32(21). 10.1091/mbc.E20-09-0568

8. Virtanen P, Gommers R, Oliphant TE, Haberland M, Reddy T, Cournapeau D, Burovski E, Peterson P, Weckesser W, Bright J et al. SciPy 1.0: fundamental algorithms for scientific computing in Python. Nat Methods. 2020;17(3):261-272. 10.1038/s41592-019-0686-2
