# Supplementary material for: Divalent nanobodies to platelet CLEC-2 can serve as agonists or antagonists
Source: Commun Biol. 2023 Apr 7;6:376. doi: 10.1038/s42003-023-04766-6 (PMC10082178; doi:10.1038/s42003-023-04766-6)
Supplement: Supplementary file 2 — Description of Additional Supplementary Data [file 42003_2023_4766_MOESM2_ESM.docx]

**Description of Additional Supplementary Files**

**File name:** Supplementary Data 1

**Description:** : The source data behind the graphs in the paper
