# Supplementary material for: Divalent nanobodies to platelet CLEC-2 can serve as agonists or antagonists
Source: Commun Biol. 2023 Apr 7;6:376. doi: 10.1038/s42003-023-04766-6 (PMC10082178; doi:10.1038/s42003-023-04766-6)
Supplement: Supplementary file 4 — Reporting Summary [file 42003_2023_4766_MOESM4_ESM.pdf]

nature portfolio

Corresponding author(s): Dr Joanne Clark
Prof Steve Watson

Last updated by author(s): Mar 16, 2023

Reporting Summary

Nature Portfolio wishes to improve the reproducibility of the work that we publish. This form provides structure for consistency and transparency in reporting. For further information on Nature Portfolio policies, see our Editorial Policies and the Editorial Policy Checklist.

Statistics

For all statistical analyses, confirm that the following items are present in the figure legend, table legend, main text, or Methods section.

n/a	Confirmed	
☐	☒	The exact sample size (*n*) for each experimental group/condition, given as a discrete number and unit of measurement
☐	☒	A statement on whether measurements were taken from distinct samples or whether the same sample was measured repeatedly
☐	☒	The statistical test(s) used AND whether they are one- or two-sided<br>*Only common tests should be described solely by name; describe more complex techniques in the Methods section.*
☒	☐	A description of all covariates tested
☐	☒	A description of any assumptions or corrections, such as tests of normality and adjustment for multiple comparisons
☐	☒	A full description of the statistical parameters including central tendency (e.g. means) or other basic estimates (e.g. regression coefficient) AND variation (e.g. standard deviation) or associated estimates of uncertainty (e.g. confidence intervals)
☒	☐	For null hypothesis testing, the test statistic (e.g. *F*, *t*, *r*) with confidence intervals, effect sizes, degrees of freedom and *P* value noted<br>*Give P values as exact values whenever suitable.*
☒	☐	For Bayesian analysis, information on the choice of priors and Markov chain Monte Carlo settings
☒	☐	For hierarchical and complex designs, identification of the appropriate level for tests and full reporting of outcomes
☒	☐	Estimates of effect sizes (e.g. Cohen's *d*, Pearson's *r*), indicating how they were calculated

Our web collection on statistics for biologists contains articles on many of the points above.

Software and code

Policy information about availability of computer code

Data collection	Zeiss Zen Black, NIS Elements 5 (Nikon Instruments), python package quickpbsa, Biacore T200 control software , ImageJ, ImageJ plugin ThunderSTORM, BD Biosciences cSampler Software
Data analysis	Biacore T200 Evaluation software, Zeiss Zen Black, python package quickpbsa, ImageJ plugin ThunderSTORM, BD Biosciences cSampler Software, PRISM v9.2.0, Microsoft Excel

For manuscripts utilizing custom algorithms or software that are central to the research but not yet described in published literature, software must be made available to editors and reviewers. We strongly encourage code deposition in a community repository (e.g. GitHub). See the Nature Portfolio guidelines for submitting code & software for further information.

Data

Policy information about availability of data

All manuscripts must include a data availability statement. This statement should provide the following information, where applicable:

- Accession codes, unique identifiers, or web links for publicly available datasets
- A description of any restrictions on data availability
- For clinical datasets or third party data, please ensure that the statement adheres to our policy

All data are available in the main text or the Supplementary Materials including Supplementary Figure 6 and Supplementary Data 1. All other data are available from the corresponding author on reasonable request

Human research participants

Policy information about studies involving human research participants and Sex and Gender in Research.

Reporting on sex and gender	This information has not been collected.
Population characteristics	This information has not been collected.
Recruitment	Healthy blood donors are volunteers and are members of our research group or other research groups within the Institute/ College.
Ethics oversight	Ethical approval for collecting blood from healthy volunteers was granted by Birmingham University Internal Ethical Review Committee (reference: ERN_11-0175).

Note that full information on the approval of the study protocol must also be provided in the manuscript.

Field-specific reporting

Please select the one below that is the best fit for your research. If you are not sure, read the appropriate sections before making your selection.

☒ Life sciences ☐ Behavioural & social sciences ☐ Ecological, evolutionary & environmental sciences

For a reference copy of the document with all sections, see nature.com/documents/nr-reporting-summary-flat.pdf

Life sciences study design

All studies must disclose on these points even when the disclosure is negative.

Sample size	The sample sizes used in this study were not predetermined. Every experiment was performed at a minimum of 3 independent experiments which is the accepted standard for this field and chosen based on previous experience.
Data exclusions	No data was excluded from the analyses.
Replication	Verification of the reproducibility of the experimental findings was confirmed by performing a minimum of 3 independent experiments of each experiment including different blood donors for every replication. The findings of all experimental replicates were consistent.
Randomization	Randomization was not required for this study as all blood donors were healthy volunteers and therefore there were no comparative experimental groups.
Blinding	Blinding was not required for this study as all blood donors were healthy volunteers and therefore there were no comparative experimental groups.

Reporting for specific materials, systems and methods

We require information from authors about some types of materials, experimental systems and methods used in many studies. Here, indicate whether each material, system or method listed is relevant to your study. If you are not sure if a list item applies to your research, read the appropriate section before selecting a response.

Materials & experimental systems

n/a	Involved in the study	
☐	☒	Antibodies
☐	☒	Eukaryotic cell lines
☒	☐	Palaeontology and archaeology
☐	☒	Animals and other organisms
☒	☐	Clinical data
☒	☐	Dual use research of concern

Methods

n/a	Involved in the study	
☒	☐	ChIP-seq
☐	☒	Flow cytometry
☒	☐	MRI-based neuroimaging

Antibodies

Antibodies used	Anti-human CLEC-2 AYP1 & AYP2 (Made in house as described [Gitz E, et al. CLEC-2 expression is maintained on activated platelets and on platelet microparticles. Blood. 2014;124(14):2262-70. 10.1182/blood-2014-05-572818]<br>Anti-6-His IgG Alexa Fluor 647 (Cat no. MA1-21315-A647, Invitrogen)

	Anti-mouse IgG Alexa Fluor 647 (Cat no. A-2123, Invitrogen)<br>Phospho-Syk (Tyr525/526) Antibody (Cat no. 2711S, Cell Signaling Technology)<br>Anti-LAT (phospho Y200) antibody (Cat no. ab68139, Abcam)<br>Anti-Phosphotyrosine Antibody, clone 4G10® (Cat no. 05-321, Merck Millipore)
Validation	Anti-human CLEC-2 AYP1 & AYP2 were validated as described here [Gitz E, et al. CLEC-2 expression is maintained on activated platelets and on platelet microparticles. Blood. 2014;124(14):2262-70. 10.1182/blood-2014-05-572818]. Each batch made is tested and validated with western blotting, flow cytometry and platelet functional assays.<br><br>All other antibodies were commercially purchased and were previously validated by the vendors and described in literature:<br>Anti-6-His IgG Alexa Fluor 647 (Cat no. MA1-21315-A647, Invitrogen): Immunogen<br>6x His synthetic peptide, Application: WB.<br>Anti-mouse IgG Alexa Fluor 647 (Cat no. A-2123, Invitrogen): Anti-Mouse secondary antibodies are affinity-purified antibodies with well-characterized specificity for mouse immunoglobulins and are useful in the detection, sorting or purification of its specified target. Immunogen: Gamma Immunoglobins Heavy and Light chains, Application: IHC, ICC/IF, Flow.<br>Phospho-Syk (Tyr525/526) Antibody (Cat no. 2711S, Cell Signaling Technology): phospho-Syk (Tyr525/526) Antibody detects endogenous levels of Syk protein only when phosphorylated at Tyr525/526 of human Syk (Tyr519/520 of mouse Syk). Antibodies are purified by protein A and peptide affinity chromatography. Application: WB.<br>Anti-LAT (phospho Y200) antibody (Cat no. ab68139, Abcam): The antibody only detects LAT phosphorylated on Tyrosine 200 of long isoform and Tyrosine 171 of short isoform. Immunogen: Synthetic peptide. This information is proprietary to Abcam and/or its suppliers. Application: WB, ICC/IF.<br>Anti-Phosphotyrosine Antibody, clone 4G10: Detects tyrosine phosphorylated proteins in all species. This unique monoclonal antibody is validated for use in IC, IH, IP, WB and is backed by hundreds of publications. Applications: ICC, IHC, IP, WB.

Eukaryotic cell lines

Policy information about cell lines and Sex and Gender in Research

Cell line source(s)	HEK293T and DT40 cells were purchased from ATCC. Podoplanin-knock out HEK293T cells were generated in-house from the parental cell lines.
Authentication	ATCC comprehensively performs authentication and quality control tests on all lots of cell lines distributed.
Mycoplasma contamination	The original cell lines were tested.
Commonly misidentified lines<br>(See ICLAC register)	No commonly misidentified lines were used in this study.

Animals and other research organisms

Policy information about studies involving animals; ARRIVE guidelines recommended for reporting animal research, and Sex and Gender in Research

Laboratory animals	WT C57BL/6 mice were purchased from Charles River Laboratories. Humanised CLEC-2 (hCLEC-2KI) mice were generated by Biocytogen on a C57Bl/6N background using CRISPR Cas9 to replace the mouse Clec1b gene with the human variant.
Wild animals	No wild animals were used in this study.
Reporting on sex	There was no sex or gender specific analysis performed in the animal studies.
Field-collected samples	This study did not involve samples collected from the field.
Ethics oversight	Animal studies were either approved by the district government of Lower Franconia or were performed in accordance with the Animal (Scientific Procedures) Act 1986 with approval of the UK Home Office under PPL PP9677279 and P06779746 granted to the University of Birmingham.

Note that full information on the approval of the study protocol must also be provided in the manuscript.

Flow Cytometry

Plots

Confirm that:

☒ The axis labels state the marker and fluorochrome used (e.g. CD4-FITC).

☒ The axis scales are clearly visible. Include numbers along axes only for bottom left plot of group (a 'group' is an analysis of identical markers).

☐ All plots are contour plots with outliers or pseudocolor plots.

☒ A numerical value for number of cells or percentage (with statistics) is provided.

Methodology

Sample preparation	Cell lines (purchased from ATCC) or human washed platelets (healthy blood donors) were stained with a primary antibody or nanobody followed by staining with a secondary antibody conjugated with a fluorophore. Further details can be found in Supplementary.
Instrument	Data were acquired using a Accuri C6 flow cytometer (BD Biosciences, USA) with cSampler Software (BD Biosciences, USA).
Software	Data was collected and analysed using cSampler Software (BD Biosciences, USA).
Cell population abundance	10,000 cells for each cell population were collected and analysed. Cell populations were gated on cell size using forward scatter (FSC) vs side scatter (SSC) to distinguish them from electronic noise. Purity of the samples was achieved by using only single cell isolated samples (E.g cell lines and purified isolated washed platelets).
Gating strategy	Cell populations were gated on cell size using forward scatter (FSC) vs side scatter (SSC) (granularity) to distinguish them from electronic noise. Control samples with no staining and secondary antibody staining only were also analysed.

☒ Tick this box to confirm that a figure exemplifying the gating strategy is provided in the Supplementary Information.
